# Genetics of vegetarianism: A genome-wide association study

**DOI:** 10.1371/journal.pone.0291305

**Published:** 2023-10-04

**Authors:** Nabeel R. Yaseen, Catriona L. K. Barnes, Lingwei Sun, Akiko Takeda, John P. Rice

**Affiliations:** 1 Department of Pathology, Feinberg School of Medicine, Northwestern University, Chicago, IL, United States of America; 2 Fios Genomics, Edinburgh, United Kingdom; 3 Department of Psychiatry, School of Medicine, Washington University in St. Louis, St. Louis, MO, United States of America; 4 Retired, St. Louis, MO, United States of America; Sun Yat-Sen University, CHINA

## Abstract

A substantial body of evidence points to the heritability of dietary preferences. While vegetarianism has been practiced for millennia in various societies, its practitioners remain a small minority of people worldwide, and the role of genetics in choosing a vegetarian diet is not well understood. Dietary choices involve an interplay between the physiologic effects of dietary items, their metabolism, and taste perception, all of which are strongly influenced by genetics. In this study, we used a genome-wide association study (GWAS) to identify loci associated with strict vegetarianism in UK Biobank participants. Comparing 5,324 strict vegetarians to 329,455 controls, we identified one SNP on chromosome 18 that is associated with vegetarianism at the genome-wide significant level (rs72884519, β = -0.11, *P* = 4.997 x 10^−8^), and an additional 201 suggestively significant variants. Four genes are associated with rs72884519: *TMEM241*, *RIOK3*, *NPC1*, and *RMC1*. Using the Functional Mapping and Annotation (FUMA) platform and the Multi-marker Analysis of GenoMic Annotation (MAGMA) tool, we identified 34 genes with a possible role in vegetarianism, 3 of which are GWAS-significant based on gene-level analysis: *RIOK3*, *RMC1*, and *NPC1*. Several of the genes associated with vegetarianism, including *TMEM241*, *NPC1*, and *RMC1*, have important functions in lipid metabolism and brain function, raising the possibility that differences in lipid metabolism and their effects on the brain may underlie the ability to subsist on a vegetarian diet. These results support a role for genetics in choosing a vegetarian diet and open the door to future studies aimed at further elucidating the physiologic pathways involved in vegetarianism.

## Introduction

Abstention from the consumption of animal flesh has been advocated for thousands of years for religious, ethical, environmental, and health-related reasons [1–8]. Eastern religious traditions that discourage meat consumption include Hinduism and Buddhism. In ancient Greece, vegetarianism was practiced as early as the 6^th^ century BC by the followers of Pythagoras and Orphism [2, 4]. During the Renaissance and Enlightenment periods several prominent personalities in Europe practiced vegetarianism, and vegetarian societies began to be established in Europe and America in the 19^th^ century [4].

While religious and ethical considerations were, and to a large extent still are, major motivations behind adopting a vegetarian diet, recent research has provided evidence for its health benefits, including lower risk of metabolic syndrome, obesity, dyslipidemias, diabetes, cardiovascular disease, and some cancers [5, 6, 8–15]. On the other hand, there is evidence that vegetarian diets can lead to nutritional deficiencies and may be associated with negative effects such as anemia, dental erosion, osteopenia, and psychological disorders [16–29].

Although vegetarianism is increasing in popularity, vegetarians remain a small minority of people worldwide; for example, in the United States, vegetarians comprise approximately 3–4% of the population [2, 4, 6, 30]. In the United Kingdom, 2.3% of adults and 1.9% of children are vegetarian according to the National Diet and Nutrition Survey reported in 2014. The true number of strict vegetarians is likely much lower, as a large proportion (approximately 48–64%) of self-identified vegetarians report consuming fish, poultry, and/or red meat [31–33]. This suggests that the desire to adhere to a vegetarian diet is overridden by environmental and/or biological constraints and raises the question of whether all humans are capable of surviving and thriving on a long-term strict vegetarian diet.

In support of a biological underpinning for vegetarianism, a large body of evidence points to a genetic component for food choice, including preferences for meat or vegetables as well as “healthy” or “unhealthy” eating patterns [34–50]. These findings indicate that differences in dietary preferences and/or needs among individuals are, at least to some extent, dictated by genetics.

To explore the contribution of genetics to vegetarianism, we utilized dietary and genetic data from the UK Biobank (https://www.ukbiobank.ac.uk/). The UK Biobank project is a prospective cohort study of approximately 500,000 individuals from the United Kingdom, aged between 40 and 69 at recruitment, that provides extensive genetic and phenotypic data, including comprehensive dietary information. Two categories of detailed dietary data were obtained from participants in the UK Biobank: (i) an initial touchscreen dietary questionnaire was administered at the recruitment session and 3 subsequent visits asking participants about their average intake of various food items over the previous year, and (ii) an online questionnaire based on 24-hour dietary recall of the previous day was administered towards the end of the recruitment session and at 4 additional instances thereafter, with invitations being emailed to participants at 3–4 monthly intervals. Genetic data were obtained from participants using two very similar genotyping arrays: a subset of about 50,000 participants were genotyped using the UK BiLEVE Axiom Array, while the vast majority of participants were genotyped using the closely related UK Biobank Axiom Array [51]. In this study we used genome-wide association (GWAS) to identify genetic loci that are associated with long-term adherence to a strict vegetarian diet among UK Biobank participants.

## Methods

### Participants

UK Biobank (UKB) is a population-based health research resource consisting of approximately 500,000 people, aged between 40 and 69 at recruitment. Phenotypic information on each participant was gathered from physical and cognitive measurements, sample collection (blood, urine, saliva), and questionnaires querying socio-demographic, lifestyle, and health-related factors. Detailed information from two questionnaires (a touchscreen questionnaire and diet by 24-hour recall questionnaire) was obtained from UKB to screen participants for inclusion in this GWAS as a vegetarianism case or control.

### Ethics statement

UKB has approval from the North West Multi-centre Research Ethics Committee (MREC) as a Research Tissue Bank (RTB). This approval means that researchers do not require separate ethical clearance and can operate under the RTB approval. The data was fully anonymized by UKB prior to our accessing them. At enrollment, electronic signed consent was obtained by UKB from all participants to use their anonymized data and samples for any health-related research and they were advised that they have the right to withdraw at any time without giving a reason and without penalty [52].

### Genotyping, imputation, and quality control

Genotyping, initial quality control (QC), phasing and imputation were conducted by UKB. The UKB QC pipeline was specifically designed to accommodate a large-scale data set containing ethnically diverse participants who were genotyped in many batches across two slightly different novel genotyping arrays, UK BiLEVE and UKB Axiom, which have over 95% common content. The genotyping, quality control and imputation of this data has been extensively described previously [51].

UK Biobank (UKB) carries out extensive sample and SNP quality control prior to imputation, which includes testing for batch effects, plate effects, extreme Hardy-Weinberg equilibrium departures, sex effects, array effects and discordance across control replicates. More information on the quality control carried out by UKB can be found in the Genotyping and quality control of UK Biobank documentation.

Pre-phasing was carried out by UK Biobank in order to derive the underlying haplotypes for each individual followed by imputation to estimate the unobserved genotypes [51, 53]. Imputation was carried out using the Haplotype Reference Consortium (HRC) data [54] as well as a merged panel consisting of the UK10K haplotype reference panel merged together with the 1000 Genomes Phase 3 reference panel [55]. Imputation was carried out with the IMPUTE4 program, which is a re-coded version of the haploid imputation functionality implemented in IMPUTE2 [51, 53]. The result of the imputation process is a dataset with 93,095,623 autosomal SNPs, short indels and large structural variants in 487,442 individuals and an additional 3,963,705 markers on the X chromosome.

In addition to the pre-imputation quality control checks, UKB also calculates metrics and provides data for a number of key variables, including relatedness, ethnicity, genetic and reported sex, heterozygosity, missingness, and genetic variables such as minor allele frequency (MAF) and INFO scores. UKB does not exclude samples based on these metrics, but provides either the metrics or a list of recommended samples to exclude based on these measurements; it is then up to the researcher to exclude any further samples. After receiving the data from UKB, additional in-house sample QC and SNP QC were then performed based on these key sample quality control metrics. The following is a summary of the Sample QC and SNP QC performed in-house; a more detailed description with figures is presented in Supporting Information files S1 and S2 Appendices, respectively.

#### Sample QC

Five sample QC metrics were assessed to produce a list of samples to exclude before downstream data analysis was performed. UK BiLEVE: samples that failed the QC procedures conducted by UKB were excluded. Samples with ethnicity other than white caucasian were excluded. To obtain an unrelated data set for analysis, individuals were excluded based on their kinship coefficient values. Any individual where the reported sex and genetic sex did not match or with sex chromosome aneuploidy was excluded. Heterozygosity and missingness outliers were excluded. Following sample QC, 161,655 unique samples were excluded from the downstream analysis. This left a total of 340,754 samples for case/control assessment and inclusion in the GWAS.

#### SNP QC

Imputed genetic data were assessed using two QC metrics. Minor allele frequency (MAF): variants with a MAF below 0.01 were excluded. Variants with an INFO score below 0.7 were excluded. Following SNP QC, 83,355,424 variants were excluded from the downstream analysis. This left a total of 9,740,199 variants for inclusion in the GWAS.

### Phenotype processing

The quality-controlled data set was split into vegetarianism cases and controls. Any sample that did not pass either the vegetarianism case or control criteria was not included in the GWAS. Following the vegetarianism screening process, 5,324 individuals were classed as vegetarianism cases and 329,455 individuals were classed as controls. Below is a summary of the phenotype processing; a detailed description of the phenotype processing methods is presented in S3 Appendix.

#### Screening vegetarianism cases

Dietary phenotype data were collected using two questionnaires: the touchscreen questionnaire, which had ~500K respondents, and the diet by 24-hour recall questionnaire, which captured ~110K of the respondents. The two questionnaires were not mutually exclusive, so both questionnaires were screened separately.

Initial exclusions were carried out based on the touchscreen questionnaire data fields asking about intake of fish, processed meat, poultry intake, beef intake, lamb/mutton intake, and pork intake. An individual was excluded if they responded "Do not know" or "Prefer not to answer" at any time point. In addition, individuals who reported eating meat within the last year and those who responded with "Prefer not to answer" were also excluded. The remaining individuals were then screened separately for the two questionnaires.

For touchscreen questionnaire screening, the data fields that were assessed in the initial exclusions were further assessed. Individuals were retained as a case if they had at least one instance within a data field answered as "Never" and every other instance was either "Never" or NA (where the individual had not completed that particular instance) within that data field. Individuals who had responded to any instance with an answer other than "Never" or NA, or had not responded to any instance within a data field were excluded.

For diet by 24-hour recall questionnaire screening, individuals who reported following a “Vegetarian” or “Vegan” diet were included. This pool of cases was then screened to exclude individuals who reported any intake of sausage, beef, pork, lamb, poultry, bacon, ham, liver, other meat, fish, seafood, and lard.

**Fig 1:**
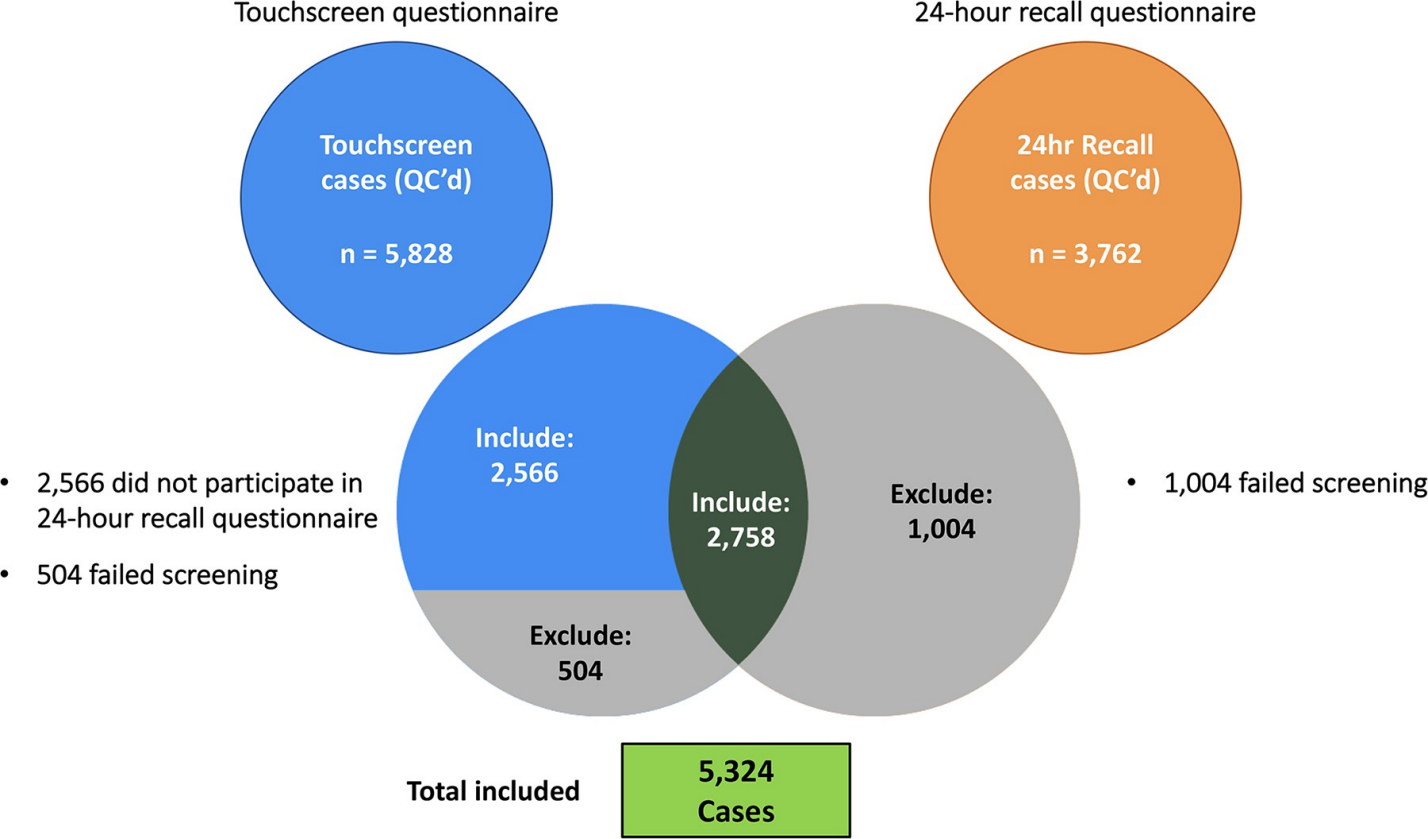
Case selection. Venn diagram showing vegetarian cases selected based on the Touchscreen and 24-hour recall questionnaires according to the criteria described in Methods section.

Screening the two separate questionnaires resulted in two overlapping pools of individuals classed as vegetarianism cases. Individuals who failed one questionnaire screening but passed the other were excluded from the total pool of cases. Individuals who passed the touchscreen questionnaire but did not fill out the 24-hour recall questionnaire were retained for inclusion within the analysis. This resulted in a final pool of 5,324 cases (Fig 1).

#### Screening vegetarianism controls

Initial vegetarianism control screening was similar to the case screening, where individuals who answered "Do not know" or "Prefer not to answer" at any time point on data fields asking about intake of fish, processed meat, poultry intake, beef intake, lamb/mutton intake, and pork intake were excluded. Remaining individuals were then screened so any individual that reported any intake on at least one occasion on any of the above data fields was retained for inclusion as a control. This resulted in a final pool of 329,455 controls.

### Statistical analysis

A detailed description of the GWAS analysis is presented in S4 Appendix. Briefly, genome-wide association analysis was performed for the vegetarianism phenotype (5,324 cases and 329,455 controls) using Scalable and Accurate Implementation of GEneralized mixed model (SAIGE). SAIGE is particularly useful for imbalanced case-control ratios and has been previously tested on UKB data [56]. A detailed description of how the program is run can be found within the SAIGE github. The models were adjusted for genetic sex (data field 22001), age when attended the assessment center (data field 21003), assessment center attended for the initial assessment visit (data field 54), principal components 1 to 20 (data field 22009) and genotyping array and batch. The threshold for genome-wide significance was set at *P* < 5 × 10^−8^ and suggestive significance was set at *P* < 5 × 10^−5^. For further analysis of the GWAS data, we used the Functional Mapping and Annotation (FUMA) platform https://fuma.ctglab.nl/ [57, 58]. Its SNP2GENE function includes the Multi-marker Analysis of GenoMic Annotation (MAGMA) tool [59]. We used FUMA version 1.5.4 and MAGMA version 1.0.8.

## Results

For the purposes of this study vegetarianism cases were defined as individuals who did not consume any animal flesh (including beef, lamb, pork, poultry, fish, and other seafood) or products derived from animal flesh, such as lard, for at least 1 year. Selection of vegetarianism cases was carried out using data from the touchscreen questionnaire, which provides diet information over the past year, and from the online 24-hour recall questionnaire, which focuses on food intake during the previous 24 hours. The touchscreen questionnaire was answered by approximately 500,000 respondents, whereas the 24-hour recall questionnaire had approximately 110,000 respondents. The touchscreen questionnaire was administered on 4 instances, and the 24-hour recall questionnaire was administered on 5 instances. A fraction of the participants responded to more than one instance of a questionnaire, and their responses for all instances were taken into consideration in determining cases and controls as described in the Methods and Supporting Information sections in detail. Screening the two separate questionnaires resulted in two overlapping pools of individuals classed as vegetarianism cases (Fig 1). While the majority of these two pools overlapped, there were some individuals who failed one questionnaire screening but passed the other. These individuals were excluded from the total pool of cases. Some individuals who passed the touchscreen questionnaire were not included within the 24-hour recall cases, as not everyone who filled out the touchscreen questionnaire also filled out the 24-hour recall questionnaire. These individuals were retained for inclusion in the analysis. This resulted in a final pool of 5,324 cases (Fig 1). Any individual that indicated any level of intake of animal flesh based on the touchscreen questionnaire on at least one instance was retained for inclusion as a control. This resulted in a final pool of 329,455 controls.

Cases and controls were compared with regard to sex, age, body mass index, and Townsend deprivation index (a measure of socioeconomic status [60]) (Table 1). Vegetarianism cases differed significantly from controls on all 4 measures. Vegetarians were more likely to be women, younger in age, have a lower body mass index, and have a higher Townsend deprivation index (lower socioeconomic status). We then performed a logistic regression analysis using all 4 variables as predictors. They each remained highly significant in the multivariate analysis. These results are in agreement with previously reported data comparing vegetarians to non-vegetarians [61–66].

**Table 1:** Characteristics of vegetarian and control populations.

	Total	Cases	Controls	p Value
**N**	334779	5324	329455	
**Sex**				<0.0001
**Male**	154585 (46%)	1788 (34%)	152797 (46%)	
**Female**	180194 (54%)	3536 (66%)	176658 (54%)	
**Age (Mean ± SD)**	56.9 ± 8.0	53.0 ± 7.9	56.9 ± 8.0	<0.0001
**Male**		53.0 ± 7.9	57.2 ± 8.1	
**Female**		53.0 ± 7.9	56.7 ± 7.9	
**Townsend (Mean ± SD)**	-1.60 ± 2.92	-1.01 ± 3.06	-1.61 ± 2.91	<0.0001
**Male**		-0.80 ± 3.20	-1.58 ± 2.97	
**Female**		-1.12 ± 2.98	-1.63 ± 2.87	
**BMI (Mean ± SD)**	27.4 ± 4.7	25.3 ± 4.5	27.4 ± 4.7	<0.0001
**Male**		25.7 ± 3.8	27.8 ± 4.2	
**Female**		25.1 ± 4.8	27.0 ± 5.1	

Vegetarian and control populations were compared for sex ratio by chi-square test, and for age, Townsend deprivation index, and body mass index (BMI) by t-test. The two populations were significantly different for all 4 variables. Logistic regression analysis was also performed using all 4 variables as predictors, and they each remained highly significant in the multivariate analysis.

GWAS was performed for vegetarianism as a binary phenotype comparing controls to vegetarians and the results are summarized below.

**Fig 2:**
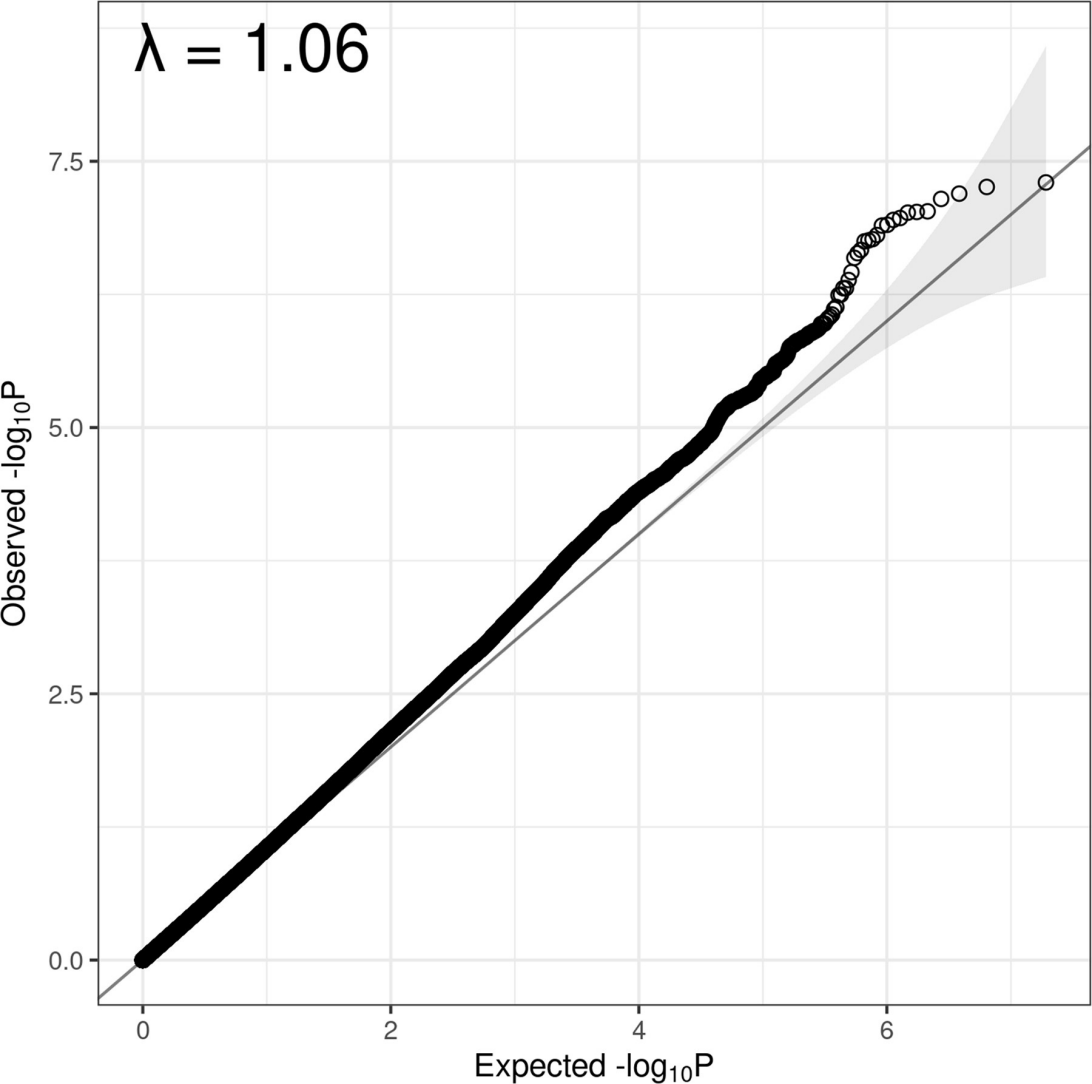
Q–Q plot. A Q–Q plot of observed versus expected -log10 (p-value). True associations are often expected for just a small minority of variants in the genome, in which case the Q–Q plot is expected to show a majority of points falling close to the expected 1:1 line, with a sharp upward curve in significance at the right-hand side of the plot (representing the true associations).

There was some mild inflation as seen in the Q-Q plot of observed versus expected -log_10_ (*P*-value), with a genomic inflation factor (lambda) of 1.06 (95% CI 1.05–1.07) (Fig 2). Inflation due to population stratification was controlled in this GWAS by excluding related individuals, providing 20 principal components to account for cryptic relatedness and using SAIGE which accounts for sample relatedness. This slight inflation may therefore be due to the imbalance of cases and controls [56], population stratification which remains after correction from SAIGE, or another, unaccounted for confounding factor.

**Fig 3:**
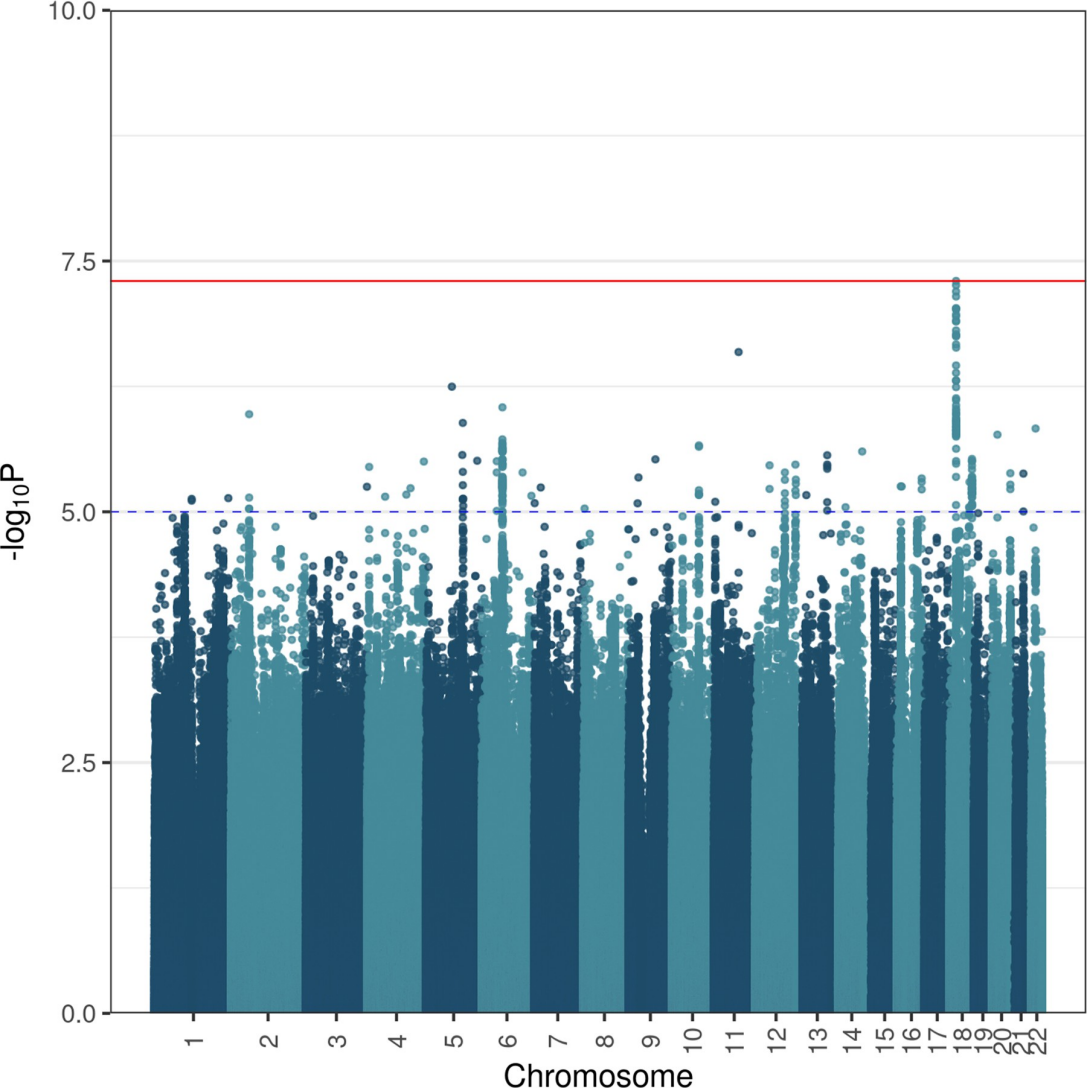
Manhattan plot. A Manhattan plot showing -log10 (p-value) for all variants as a function of position on the chromosomes. Sections of the genome showing association with vegetarianism cases can be identified as peaks within this plot. Due to linkage disequilibrium, adjacent variants are expected to show similar associations, and as a result, peaks of statistical significance may extend over several variants. The blue horizontal dashed line at -log10(1x10-5) represents the statistical threshold of suggestive association, while the red horizontal line at -log10(5x10-8) represents the statistical threshold of genome-wide significance.

**Fig 4:**
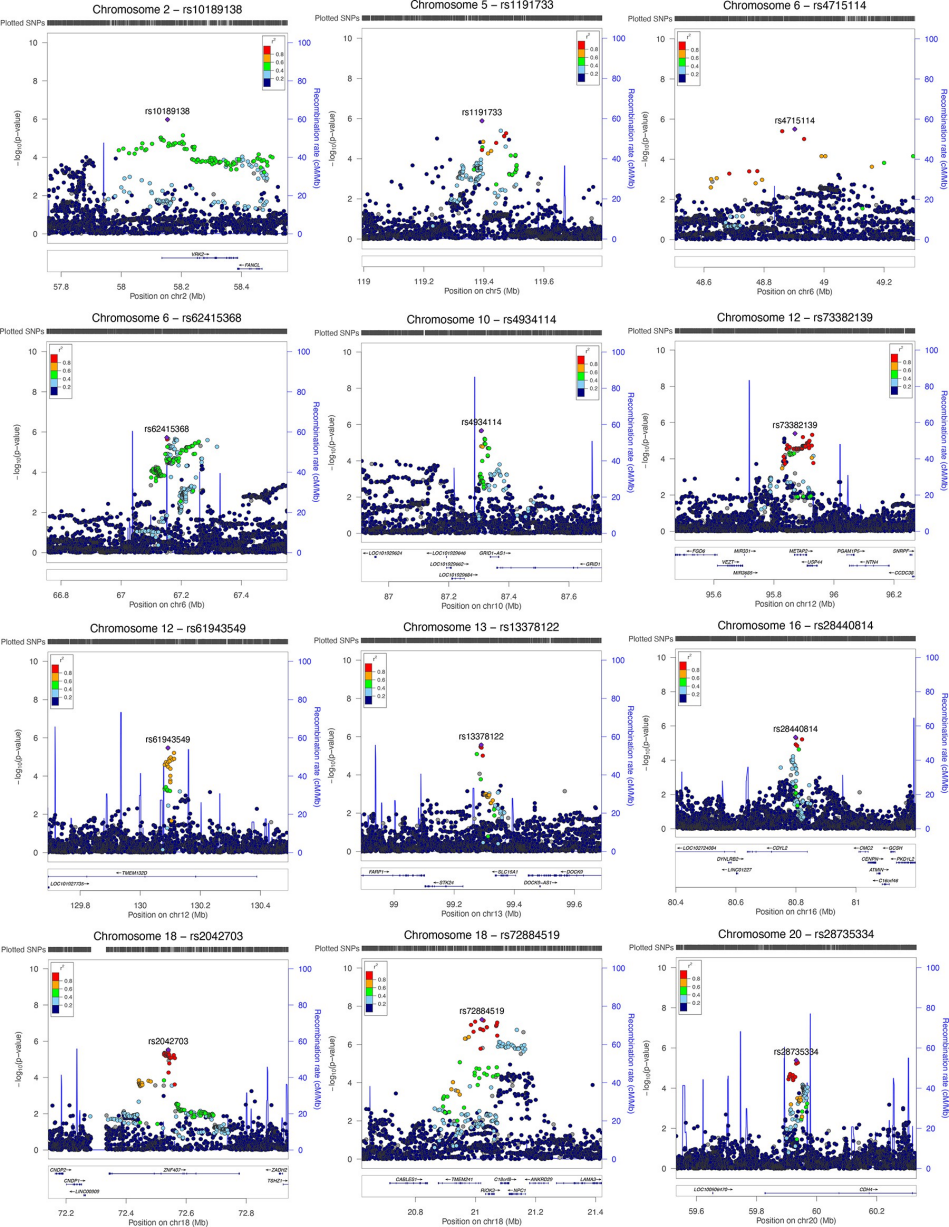
LocusZoom plots. LocusZoom plots are used to visualize GWAS results for 12 loci of interest. These loci include the one on chromosome 18 with the GWAS-significant SNP rs72884519, as well as loci with groups of 3 or more suggestively significant SNPs. LocusZoom plots display regional information including the strength and extent of the association signal relative to genomic position, local linkage disequilibrium (LD) and recombination patterns, and the positions of genes in the region. The x-axis shows the genomic position. The left-hand y-axis shows the -log10(p-value) as reported in S1 Table. The right-hand y-axis, which corresponds to the blue peaks within the plot, shows the recombination rate (cMMb) of that region of the chromosome. In each plot the variant with the lowest p-value in that region is shown as a purple diamond, while the other SNPs in the region are shown as small circles, color-coded to indicate their degree of LD with that variant. Based on the LD score metric r2. An r2 of 0.8 would mean that the two SNPs are co-inherited roughly 80% of the time. In cases where there was no LD information available for a SNP (as is the case for chromosome 6 SNP rs62417319), the next most significant SNP has been plotted (rs62415368). Below the plot is the location of genes within the region, if present. These plots were generated using the original LocusZoom tool using the default settings. Summary statistics for the chromosome corresponding to the SNP of interest were used as input.

At the genome-wide significance threshold of 5 x 10^−8^, one SNP on chromosome 18 was found to be significant: rs72884519, (β = -0.11, *P* = 4.997 x 10^−8^). A number of suggestively significant SNPs in high linkage disequilibrium (LD) with rs72884519 were identified in the surrounding area, within genes *RIOK3*, *NPC1*, *RMC1* (C18orf8), and *TMEM241* (Figs 3 and 4, S1 Table).

Several other loci with groups of suggestively significant variants were identified, for a total of 202 significant and suggestively significant variants (Figs 3 and 4, S1 Table). An additional 7 genes associated with these loci contain at least one suggestively significant variant each (Fig 4 and S1 Table). This brings the total of genes that may contribute to the vegetarianism phenotype to 11: *RIOK3*, *NPC1*, *RMC1* (C18orf8), *TMEM241*, *VRK2*, *TMEM132D*, *METAP2*, *USP44*, *CDYL2*, *ZNF407*, *and CDH4*. A brief overview of what is known about the functions of these genes based on clinical and experimental data is provided in S5 Appendix, and a summary of traits associated with these genes identified by previous GWAS studies is provided in S2 Table.

**Fig 5:**
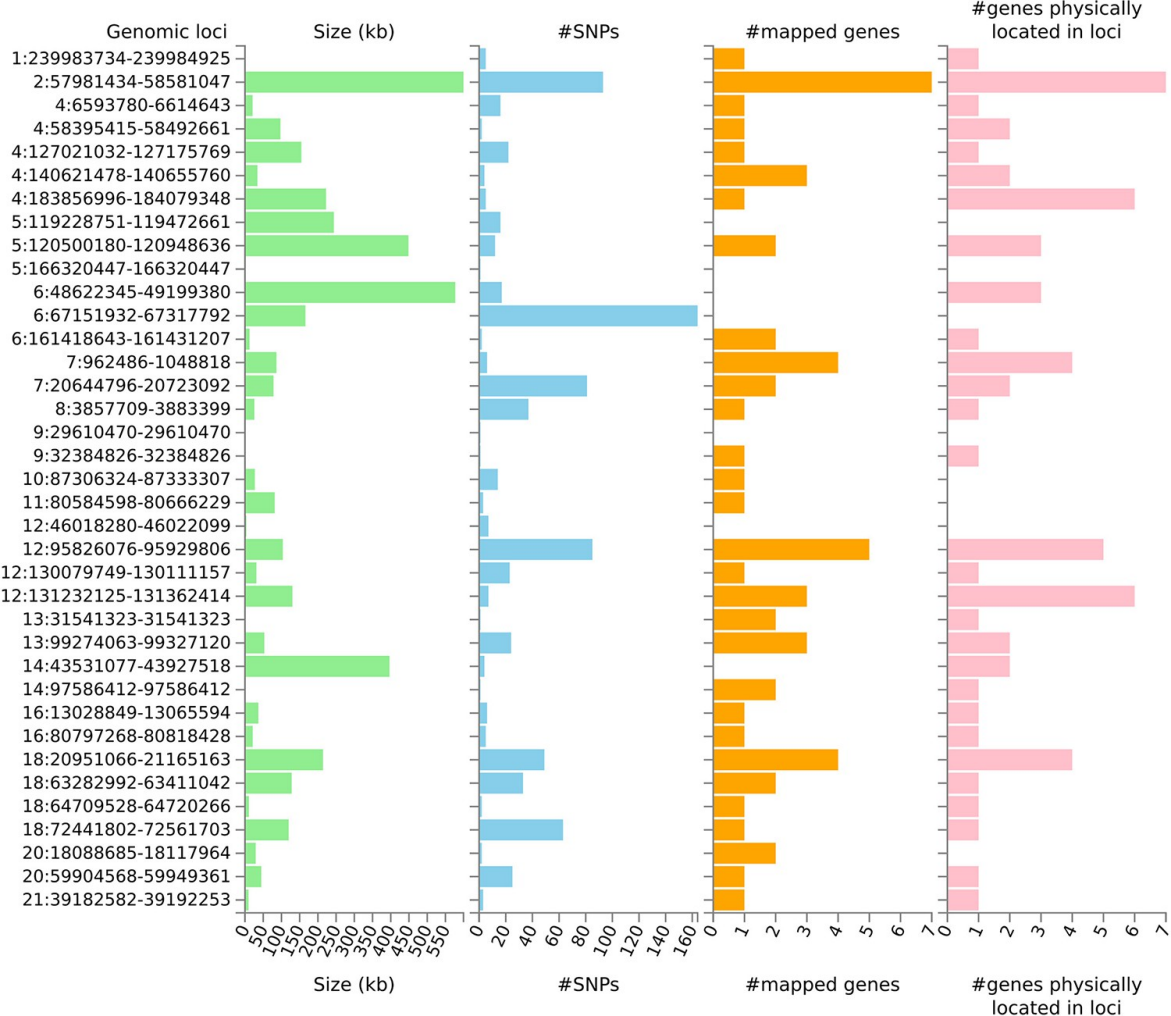
Summary of genomic risk loci. The histograms show summary results for each genomic locus identified by FUMA analysis. See text.

Further analysis of the GWAS data was carried out using the FUMA platform, which includes the MAGMA tool [57–59]. FUMA identified 37 genomic risk loci for vegetarianism and mapped 842 candidate SNPs and 59 genes to those loci (Fig 5 and S2–S4 Tables).

**Fig 6:**
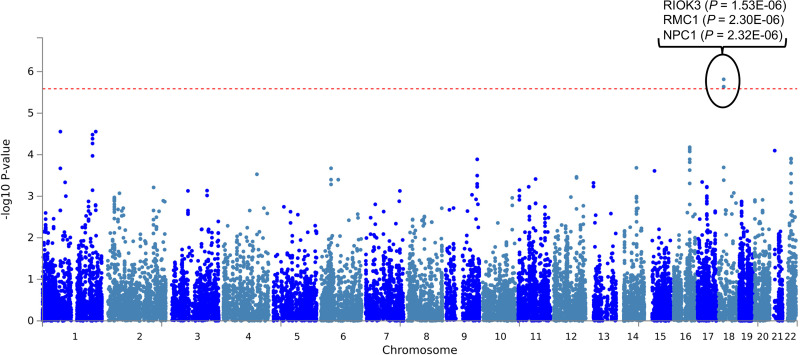
Gene-based Manhattan plot. Gene-based GWAS analysis was carried out using the MAGMA tool. The Manhattan plot shows -log10 (p-value) for all genes as a function of position on the chromosomes. Input SNPs were mapped to 19295 protein coding genes. Genome wide significance (red dashed line in the plot) was defined at P = 0.05/19295 = 2.591e-6.

Gene-level GWAS analysis was carried out by MAGMA, which identified 3 genes with GWAS-significant p-values that clearly stand out from the rest: *RIOK3*, *RMC1*, and *NPC1* (Fig 6). The full MAGMA gene-level results are shown in S6 Table. Intersecting the mapped gene list with the MAGMA-generated gene list yields 34 genes with possible roles in vegetarianism, listed in S7 Table, sorted by MAGMA gene p-value.

**Fig 7:**
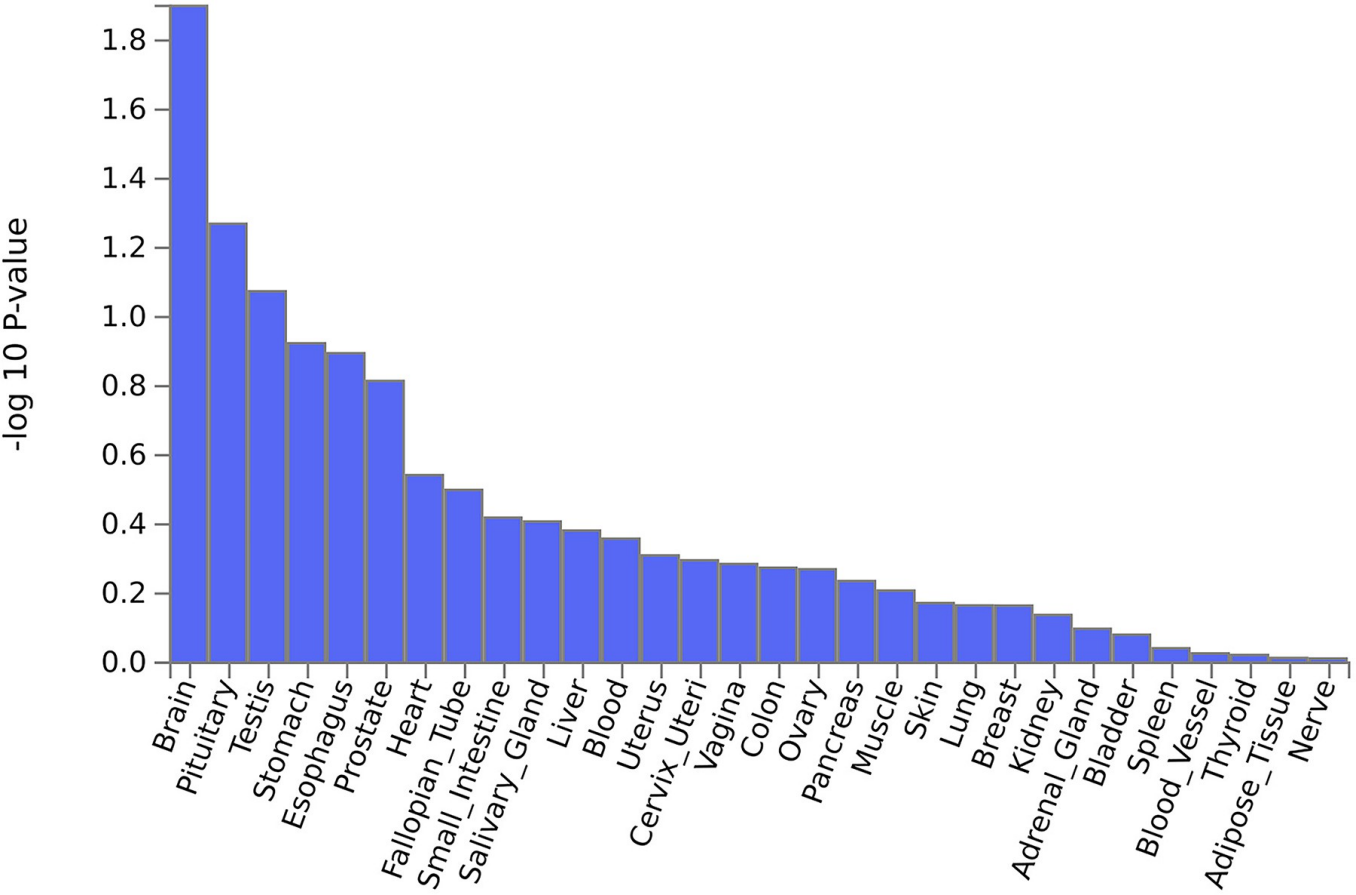
MAGMA tissue expression analysis: GTEx v8 30 general tissue types. To identify tissue specificity of the phenotype, FUMA performs MAGMA gene-property analyses to test relationships between tissue specific gene expression profiles and phenotype-gene associations. GTEx eQTL v8 contains 30 general tissue types.

Of the 3 GWAS-significant genes, *NPC1* is notable for being associated with SNPs that have the highest probability of being functionally relevant, with CADD score of 18.56 (rs1788799), RegulomeDB score of 1f (rs1624695), and commonChrState score of 1 (rs1623003) (S4 Table). The RegulomeDB parameter is used to predict the functionality of variants [67]; the CADD parameter prioritizes functional, deleterious and pathogenic variants [68]; and the commonChrState parameter indicates the most common 15-core chromatin state across 127 tissue/cell types [69]. *NPC1* (NPC Intracellular Cholesterol Transporter 1) encodes a large protein that resides in the membrane of endosomes and lysosomes and mediates intracellular trafficking of cholesterol and glycolipids [70–78]. Recent data indicate that activation of Rab7 by a trimeric GEF complex that includes RMC1, is required for lysosomal NPC1-dependent cholesterol export [79]. Mutations in *NPC1* are responsible for 95% of cases of Niemann-Pick disease type C, a lysosomal storage disease characterized by intracellular accumulation of cholesterol and glycosphingolipids in various tissues, with progressive neurological disease being the most significant clinical manifestation [80, 81]. As further discussed below, these findings suggest that the genetic contribution to vegetarianism may be related to lipid metabolism and its role in brain function. This notion is supported by MAGMA tissue expression analysis showing that vegetarianism-associated variants may preferentially regulate gene expression in the brain (Fig 7). Additional support for this hypothesis is provided by FUMA analysis showing that SNPs associated with vegetarianism are also associated with several other traits in the GWAS Catalog that pertain to lipid metabolism and brain function (S8 Table).

## Discussion

Numerous studies point to a significant genetic influence on food choice [34–48]. In this study we used GWAS to identify loci associated with long-term strict vegetarianism. Using data from UK Biobank, we identified one GWAS-significant SNP (rs72884519) and many suggestively significant variants (S1 Table, Figs 3 and 4). Several of these suggestively significant variants are in high LD with rs72884519 (S1 Table, Figs 3 and 4), indicating that the result is not an artifact. The finding of only one GWAS-significant SNP could be due either to a lack of power, or lack of polygenicity. To investigate this, we performed a power calculation with CaTS (http://csg.sph.umich.edu//abecasis/CaTS). The power for a common SNP of frequency 50%, p-value of 10^−7^ and with a genotypic relative risk of 1.5 is 97% and 91% for a multiplicative and additive model, respectively, and 32% and 25% for a risk of 1.1. We chose a frequency of 50% since the power is much lower for rare SNPs, and a p-value of 10^−7^ to increase power. If there were hundreds of common causal SNPs, we would expect many more GWAS-significant ones even with modest power. We also performed LD score regression [82] and estimated the heritability to be 1.5%, with a 95% confidence interval of .9% - 15.2%. The intercept was estimated to be 1.01, indicating lack of population stratification. Overall, these data would support the hypothesis that our results may be due to a lack of polygenicity. More studies with larger sample sizes are needed to address this.

The significant and suggestively significant variants we identified are associated with 11 genes that may contribute to the vegetarianism phenotype. The functions of these genes are summarized in S5 Appendix, and their associations based on previous GWAS studies are summarized in S2 Table. Those 11 genes were also among a set of 34 genes with possible roles in vegetarianism identified by MAGMA and FUMA analysis (S7 Table). Gene-level GWAS analysis by MAGMA showed that 3 of these genes, *RIOK3*, *RMC1*, and *NPC1*, are associated with vegetarianism at the GWAS-significant level (Fig 6 and S7 Table). In addition, previous GWAS studies have implicated some of these genes, including *RIOK3*, *NPC1*, *RMC1*, and *VRK2*, in dietary choices, lending further support to their role in vegetarianism (S2 Table).

The mechanisms by which genetic variants influence dietary choices involve an interplay between metabolism, physiologic effects, and taste perception. The levels of liking and consumption of dietary items are influenced by taste perception [83]. However, taste perception of a particular dietary item can be strongly influenced by its physiologic effects, which in turn are dictated by its metabolism. For example, while perception of bitterness impacts the levels of intake of caffeine [84, 85], there is evidence that the genetically determined rate of caffeine metabolism, which in turn determines its physiologic effects, influences both bitterness perception and the level of intake of caffeine, such that a low level of caffeine metabolism is associated with greater sensitivity to bitterness and a lower level of coffee consumption and vice versa [86–88]. Similarly, consumption of alcohol is strongly affected by genetic differences in enzymes involved in ethanol metabolism. Ethanol is converted to acetaldehyde by alcohol dehydrogenase (ADH1B) and acetaldehyde is further metabolized by aldehyde dehydrogenase 2 (ALDH2) to acetate. Since acetaldehyde has aversive effects, variants of ADH1B and ALDH2 that increase the levels of acetaldehyde are associated with aversion to alcohol, whereas variants associated with decreased acetaldehyde are associated with increased alcohol consumption and alcohol dependence [89].

It is therefore possible that choosing between a vegetarian and a non-vegetarian diet may also be dictated by individual differences in both metabolism and taste preferences. One study points to a genetic difference in lipid metabolism between vegetarians and non-vegetarians that appears to represent an adaptation to a vegetarian diet [90]. Long-chain polyunsaturated fatty acids (LCPUFA) are components of membrane phospholipids that play important roles in signal transduction; they are obtained from animal food sources or synthesized endogenously through a pathway that involves the fatty acid desaturases FADS1 and FADS2. An insertion in the *FADS2* gene that increases endogenous LCPUFA synthesis appears to have been selected for in vegetarian populations [90].

Interestingly, 2 of the 3 GWAS-significant genes associated with vegetarianism, *NPC1* and *RMC1*, also function in lipid metabolism. NPC1 mediates intracellular trafficking of cholesterol and glycolipids [70–78], and mutations in *NPC1* are responsible for 95% of cases of Niemann-Pick disease type C, a lysosomal storage disease characterized by intracellular accumulation of cholesterol and glycosphingolipids in various tissues [80, 81]. As discussed above, SNPs associated with *NPC1* have a high likelihood of being functionally relevant (S4 Table). RMC1 is necessary for cellular LDL-cholesterol uptake, and NPC1-dependent lysosomal cholesterol export [79, 91], and RMC1-deficient cells exhibit severe defects in LDL trafficking, with swelling of the late endosomal/lysosomal compartment and marked lysosomal cholesterol accumulation [79]. Another of the genes associated with vegetarianism, *TMEM241*, which has the 4^th^ lowest p-value (S7 Table), is homologous to yeast *VRG4* that is involved in glycoprotein modification and mannosylation of sphingolipids [92], and decreased expression of *TMEM241* is associated with increased serum triglyceride levels in a Mexican population [93]. In addition, previous GWAS studies have shown that several vegetarianism-associated genes that we identified, including *TMEM241*, *NPC1*, *RMC1*, *RIOK3*, *VRK2*, and *TMEM132D*, are associated with markers of lipid metabolism and obesity such as triglyceride levels, LDL and HDL cholesterol levels, BMI, waist circumference, body fat, and others (S2 and S8 Tables).

Taken together, these data raise the possibility that differences in lipid metabolism may underlie the choice between a vegetarian and non-vegetarian diet. Lipid profiles of foods from animal sources are significantly different from those of plant sources, particularly with regard to complex lipids such as sphingolipids; and recent studies have brought attention to the importance of dietary sphingolipids in health [94–97]. In this regard it is of interest to note that complex lipids, particularly sphingolipids, play critical roles in nervous system development and function [98, 99], and that most of the vegetarianism-associated genes identified in this study have also been linked to psychological and neurological traits and diseases including Niemann-Pick disease, Alzheimer’s disease, epilepsy, anxiety, depression, alcoholism, schizophrenia, autism spectrum disorder, cognitive performance and others (see S2 and S8 Tables). For example, *NPC1* mutations are responsible for the vast majority of cases of the neurological disorder Niemann-Pick disease type C [80, 81], and GWAS studies have shown links between *NPC1* and Alzheimer’s disease [100]. Similarly, GWAS studies have linked *RIOK3*, *RMC1*, *NPC1*, *VRK2*, and *CDH4*, to educational attainment, cognitive performance, and/or alcohol consumption (S2 and S8 Tables). In addition, GWAS studies have shown associations between *VRK2* and size of the cerebral cortex, epilepsy, depression, bipolar disorder, anorexia nervosa, schizophrenia, autism spectrum disorder, and post-traumatic stress disorder (S2 Table). Of particular interest is the reported link between *VRK2* and anorexia nervosa [101]. Our data show that strict long-term vegetarianism is approximately twice as frequent in females (Table 1). Eating disorders are also more common in females and several studies suggest a link between vegetarianism and eating disorders [28, 102–108]. Thus, a better understanding of the metabolic underpinnings of vegetarianism may shed light on the mechanisms underlying eating disorders, particularly anorexia nervosa. The roles that the genes we identified might play in neural function and in dietary choices remain to be determined. Based on the known functions and associations of some of these genes (S5 Appendix and, S2 and S8 Tables), one possibility is that the mechanisms involved may be related to the metabolism of complex lipids and its role in brain function.

Limitations of this study include the "healthy volunteer" selection bias in UK Biobank participants, who tend to be female, older, healthier, and of better socioeconomic status than the general population [109]. Our study was focused on one population of white UK persons in order to avoid confounding by ethnicity; it would be of interest for future studies to determine whether our findings can be replicated in other white and non-white populations. In addition, UK Biobank dietary data rely on self-reporting of prior food consumption. An interventional study would not be practical with a population of this size; however, once we have a better understanding of the gene variants associated with vegetarianism, it may become possible to carry out such a study prospectively to determine if a particular genetic signature can predict an individual’s ability to adhere to a vegetarian diet. Finally, while our study is focused on the genetic contribution to strict long-term vegetarianism, dietary choices are not determined by genetics alone. Environmental factors and multiple disorders, and the medical interventions used to treat them, can impact dietary choices. Further studies are needed to determine the contributions of the genes we identified to the interplay between dietary choices and disorders of neural function and lipid metabolism.

In conclusion, our findings add to the existing body of data pointing to the genetic contribution to dietary choices and raise the possibility that lipid metabolism and its role in brain function may play a role in the ability to subsist long-term on a strict vegetarian diet. Further studies are required to determine which of the genes we identified play an important role in choosing a vegetarian diet; what particular variants of those genes underlie the vegetarianism phenotype and the mechanisms by which they contribute to this phenotype. It is tempting to speculate that meat may contain unique lipid components that vegetarians are able to adequately synthesize endogenously, whereas others need to obtain them from a meat-containing diet. A better understanding of the physiologic pathways involved in choosing a vegetarian diet is needed in order to design more effective dietary recommendations and interventions.

## Supporting information

S1 Appendix Sample QC. (PDF)

S2 Appendix SNP QC. (PDF)

S3 Appendix Phenotype processing methods. (PDF)

S4 Appendix GWAS methods. (PDF)

S5 Appendix Genes with possible roles in vegetarianism. (PDF)

S1 Table Vegetarianism-associated variants. (XLSX)

S2 Table Other genome-wide associations of vegetarianism-associated genes. (DOCX)

S3 Table FUMA genomic risk loci. (XLSX)

S4 Table FUMA candidate SNPs. (XLSX)

S5 Table FUMA mapped genes. (XLSX)

S6 Table MAGMA genes. (XLSX)

S7 Table FUMA-MAGMA genes intersect. (XLSX)

S8 Table FUMA GWAS catalog associations. (XLSX)
