# Supplementary material for: Genetics of vegetarianism: A genome-wide association study
Source: PLoS One. 2023 Oct 4;18(10):e0291305. doi: 10.1371/journal.pone.0291305 (PMC10550162; doi:10.1371/journal.pone.0291305)
Supplement: S1 Appendix — (PDF) [file pone.0291305.s001.pdf]

Sample QC

UKBiLEVE sample quality control

The UK BiLEVE array was used to genotype the first 50,000 individuals in UK Biobank (UKB) for quality control passes or fails. A number of quality control steps were carried out by the University of Leicester (the location of the UK BiLEVE project) which examined:

- Sex mismatches: samples failed if the submitted gender was different from the gender inferred from the sex chromosomes.
- Final call rate: samples failed if they had a call rate less than 95%.
- Heterozygosity: samples failed if they had a heterozygosity greater than the 3rd standard deviation from the mean heterozygosity.
- Unintended duplicate samples: samples failed if they had over 98% of alleles shared identical by descent.
- Ancestry principal components: samples failed if they had principal component values greater than 10 standard deviations from the mean on the first 10 principal components.

This resulted in a list of 549 fails (individuals to exclude) and 49,438 passes, which were summarized in data field 22051. These 549 individuals were excluded from downstream analysis in this project.

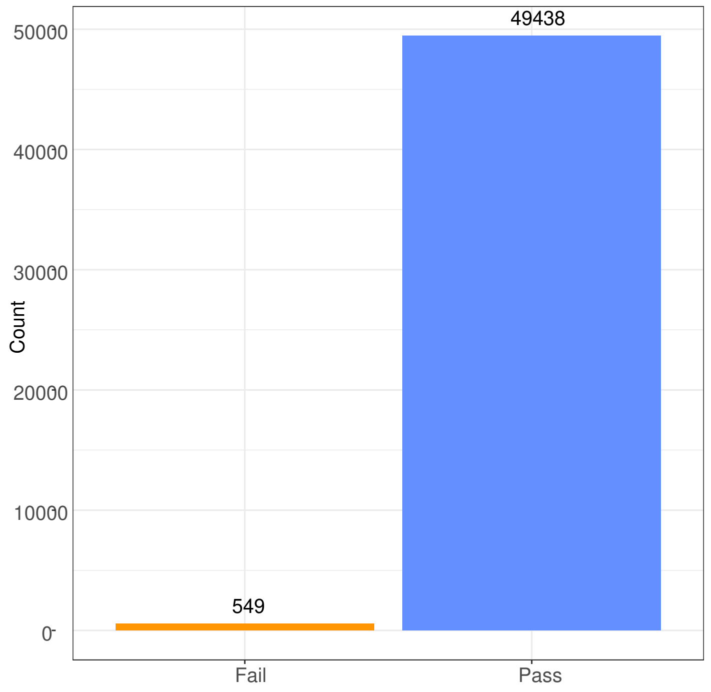

UK BiLEVE sample quality control
This plot summarizes samples that passed or failed the UK BiLEVE sample quality control procedures, which were carried out on the first 50,000 individuals genotyped on the UK BiLEVE array. Only those genotyped on this array are included in the plot. 549 individuals were marked as a fail and were subsequently excluded from downstream analysis in this project.

Ethnicity
UKB contains individuals from a variety of ethnic backgrounds, including "White", "Mixed", "Asian or Asian British", "Black or Black British" and "Chinese" (data field 21000). Within a GWAS, it is important to analyze individuals originating from the same ancestral group to avoid confounding. Therefore, 92,858 individuals who were not classed as "caucasian" under [data field 22006] were excluded from downstream analysis in this project. Please note that a number of individuals self-classified as "White" under data field 21000, however these were marked for removal by UKB under [data field 22006] as these individuals had mixed genetic ancestry and could therefore not be classed under data field 22006 as "caucasian".

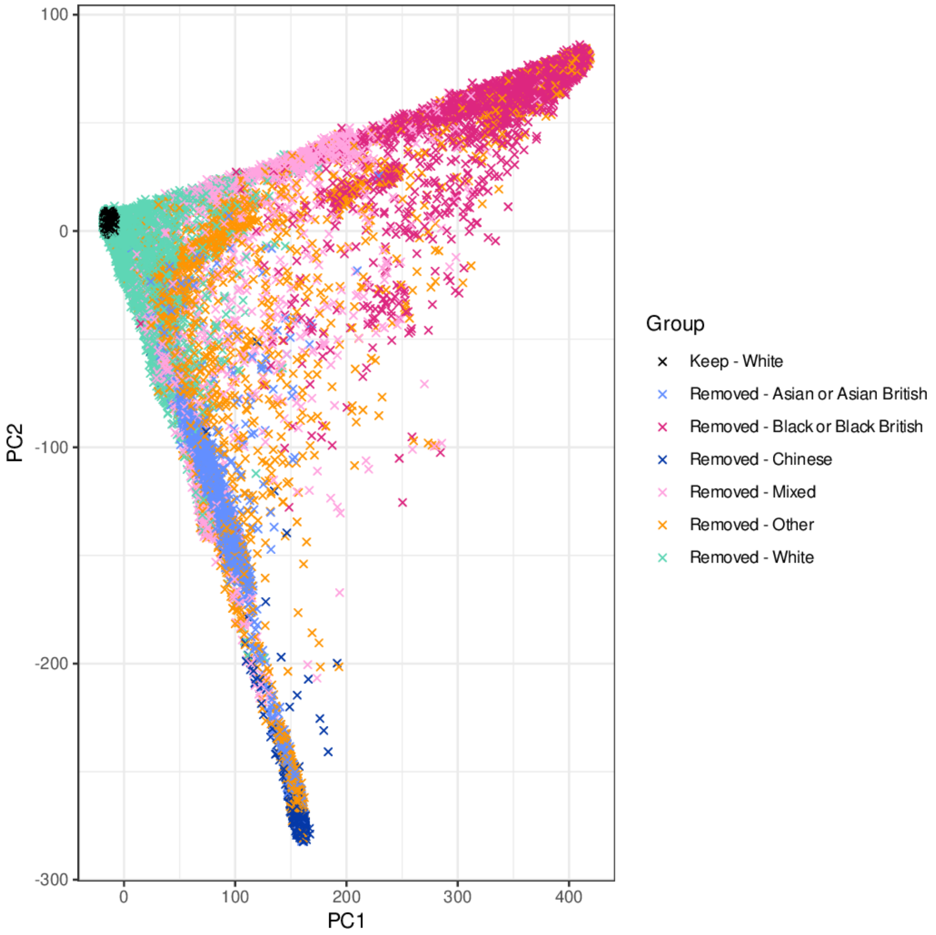

Principal component plot of ethnicity
Each point represents a UKB participant (n = 502,409 samples) and is placed according to the principal component scores for the first two principal components. Colors indicate the self-reported ethnic background of each individual, and indicate whether that group was retained or excluded. The principal component values were obtained from data field 22009 while the ethnic groups were obtained from data field 21000. Individuals were grouped according to their top level ethnic grouping: "White" (n = 472,680), "Asian or Asian British" (n = 9,885), "Black or Black British" (n = 8,065), Chinese (n = 1,571), Mixed (n = 2,985) or Other (n = 7,223). If an individual listed two "Level 1" ethnicities originating from different top level groupings, they were classed as "Mixed". The "Other" category includes those who answered "Other", "Do not know" or "Prefer not to answer" as their ethnicity. Individuals were classed as "Keep" for subsequent analyses if they were classed as "caucasian" under data field 22006 (n = 409,551). Individuals were classed as "Removed" if they were not classed as "caucasian" under data field 22006 (n = 92,858).

Relatedness

UKB calculated kinship coefficients for all pairs of samples (n = ~ 11,250,000,000) using KING's robust estimator. Kinship coefficients for all 3rd degree relatives and closer were provided by UKB within a relatedness file (n = 107,147), and shown in the below plot. Using these data, a list of individuals to exclude from UKB was generated to retain a maximum independent data set, where no two individuals had a kinship coefficient greater than 0.0442. To maximize the number of vegetarianism cases retained in the data set, any pair that contained a subject present within the vegetarianism phenotype was first assessed and the non-vegetarianism subject within the pair was selected for exclusion. In pairs where both samples were present within the vegetarianism cases, a sample was selected at random to exclude. This continued until no pairs containing a vegetarianism case remained. For the remaining relatedness pairings, samples were excluded iteratively with individuals related to the highest number of other individuals selected first, to provide a maximal number of unrelated samples. A total of 74,752 individuals were selected for exclusion.

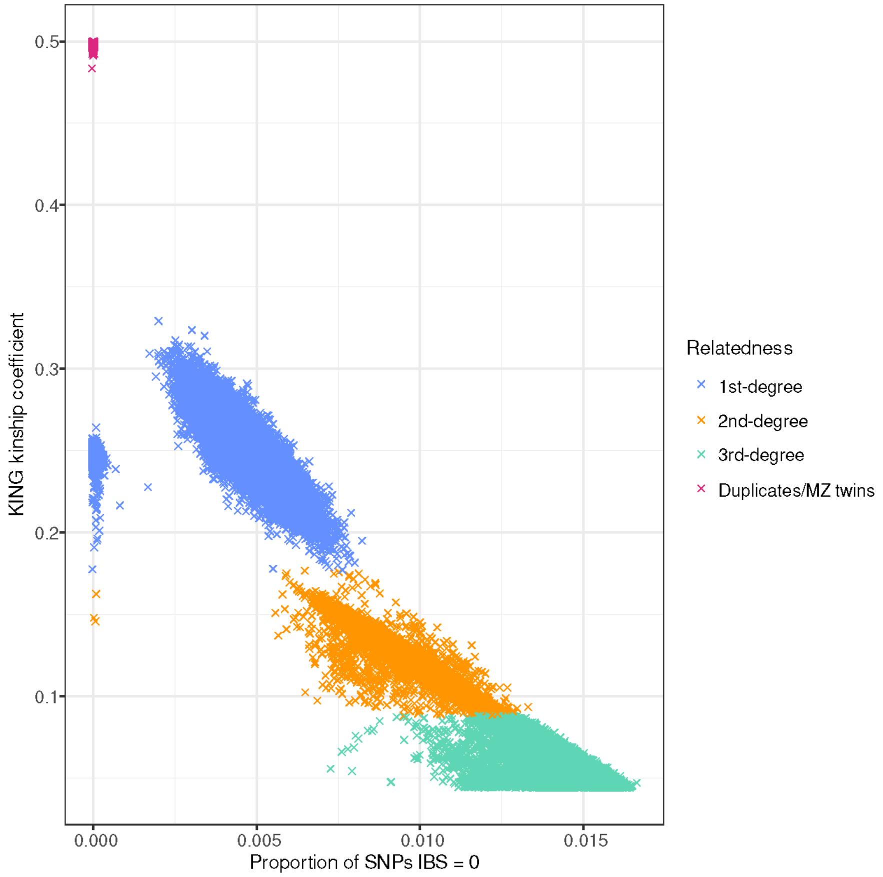

Relatedness in UKB data

This plot summarizes close relationships within the UKB data (n = 107,147 relationship pairs). Each point represents a pair of related individuals and the colors indicate the degree of relatedness: monozygotic twins in dark pink (in the upper left corner), 1st, 2nd and 3rd degree relatives in blue, orange and turquoise, respectively. There are two groups of 1st degree relatives: parent-child pairs (blue cluster in the far left) and full siblings (blue center cluster). For all pairs, the x-axis shows the proportion of zero identity-by-state (IBS0), defined as the proportion of SNPs at which one sample carries the minor homozygote and the other sample carries the major homozygote, so that they share no alleles (i.e. the individuals have two different alleles). The y-axis shows the kinship coefficient, defined as the probability that two alleles sampled at random (one from each individual) are identical by descent.

Sex mismatch and sex chromosome aneuploidy

UKB provided two measures of sex: reported sex (data field 31, n = 502,414) and genetic sex (data field 22001, n = 488,176). Reported sex was acquired from the central registry at recruitment, but in some cases was updated by the participant. Genetic sex was calculated from the genotyping analysis by UKB. Using this information, any individual where the reported sex and genetic sex did not match were excluded (n = 372). Individuals for whom genetic sex was not provided (n = 14,238) were retained for further QC processing. In addition to those samples with a sex mismatch, a further 470 samples with sex chromosome aneuploidy (data field 22019) were excluded.

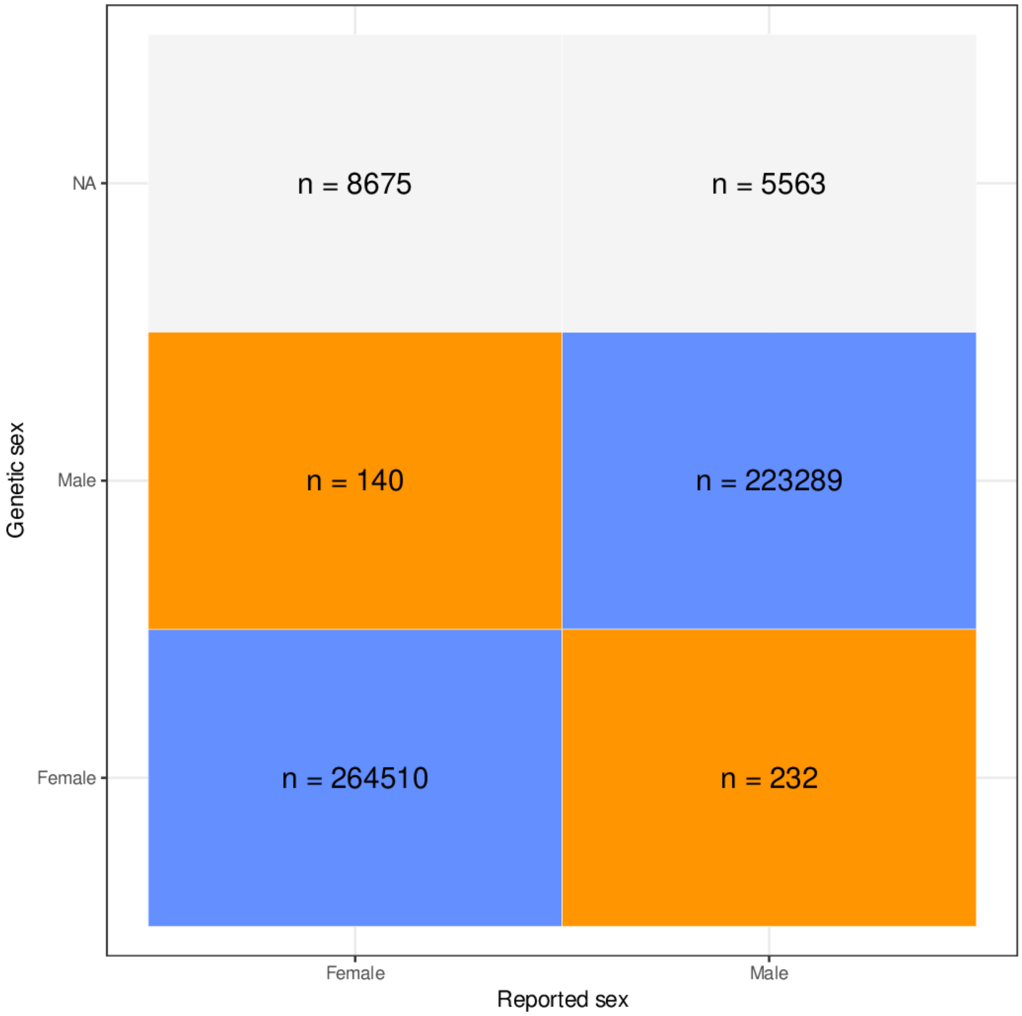

Sex concordance

This plot summarizes sex concordance between two measures of sex in UKB, reported sex and genetic sex (n = 502,409). Mismatches between reported and genetic sex are highlighted in orange; this may be due to sample mishandling or other kinds of clerical error, transgender individuals, or instances of real (but rare) genetic variation. A number of individuals have a reported sex but no genetic sex information was provided by UKB (colored in grey). Individuals included in the orange boxes were excluded, while individuals within the blue or grey boxes were retained.

Heterozygosity and missingness
Heterozygosity, the fraction of non-missing markers that are called as heterozygous, and missingness, the proportion of genotypes not called, are two metrics used to identify poor quality samples. Both metrics were computed by UKB using a set of high-quality autosomal markers (n = 605,876), and reported within fields 22003 for heterozygosity and 22005 for missingness. UKB assessed both metrics, taking into account genetic factors such as population structure, ethnicity and runs of homozygosity, to determine which samples were of poor quality. Samples with unusually high heterozygosity or a greater than 5% missing rate were flagged as outliers (data fields 22027 and 22010, n = 1,357), and were excluded from downstream analysis in this project.

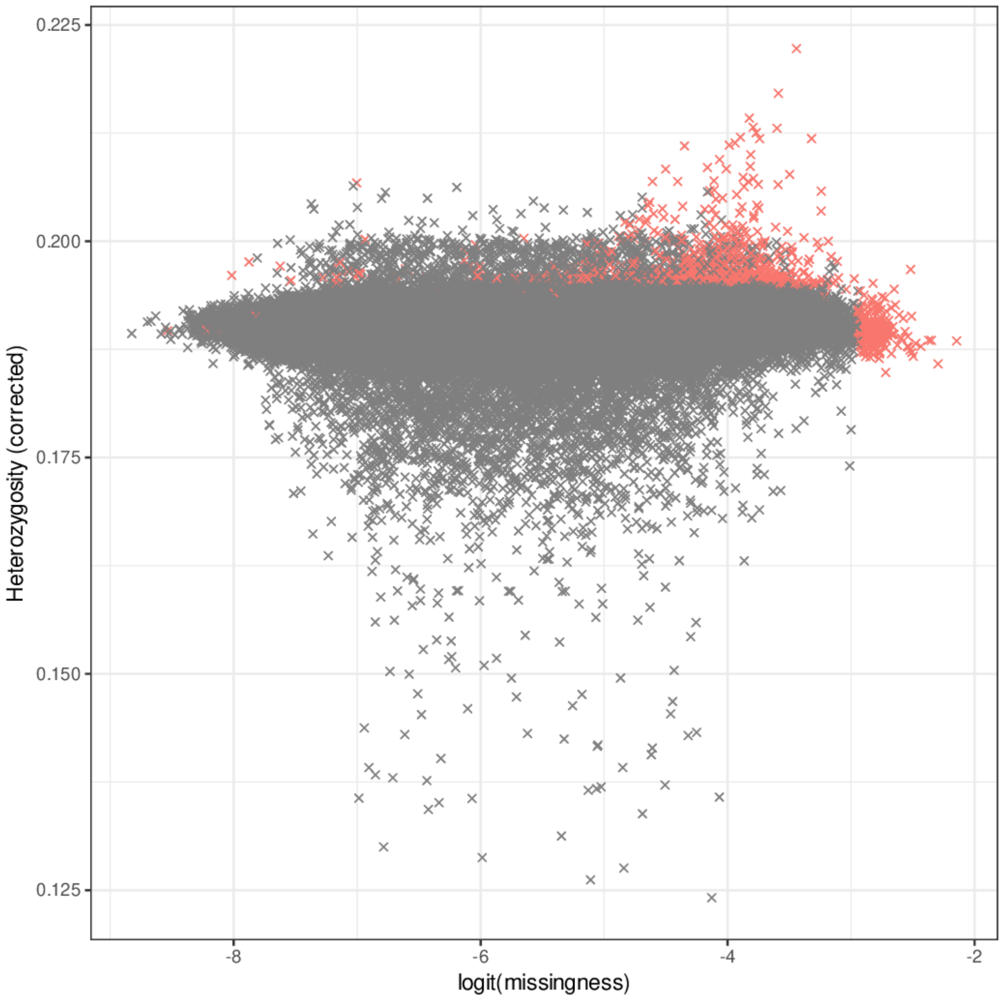

Excess heterozygosity and missingness
This plot shows ancestry-corrected heterozygosity (the proportion of autosomal heterozygous calls, data field 22004) on the y-axis and logit-transformed missingness (the proportion of genotypes not called, data field 22005) on the x-axis. Each point represents a UKB participant (n = 488,173). Subjects flagged for removal by UKB have been colored in red (data field 22027 and data field 22010). The heterozygosity values were corrected by UKB using four genetic principal component values to correct for systematic differences from population structure. The logit transformation was applied to normalize the missingness values, and is defined as logit(x) = log(x/(1-x)).
