# Supplementary material for: Genetics of vegetarianism: A genome-wide association study
Source: PLoS One. 2023 Oct 4;18(10):e0291305. doi: 10.1371/journal.pone.0291305 (PMC10550162; doi:10.1371/journal.pone.0291305)
Supplement: S2 Appendix — (PDF) [file pone.0291305.s002.pdf]

SNP QC

Minor allele frequency
A filtering criterion was applied to the minor allele frequency (MAF) of variants within the UKB data. MAF was provided by UKB for all variants. MAF is the frequency at which the second most common allele occurs in a given population. If the frequency of the minor allele is too low within a population, there will not be adequate power to make meaningful statistical statements. Therefore, a MAF threshold of < 0.01 was applied to the data, resulting in 83,160,917 variants with a MAF of less than 1% being excluded from downstream analysis.

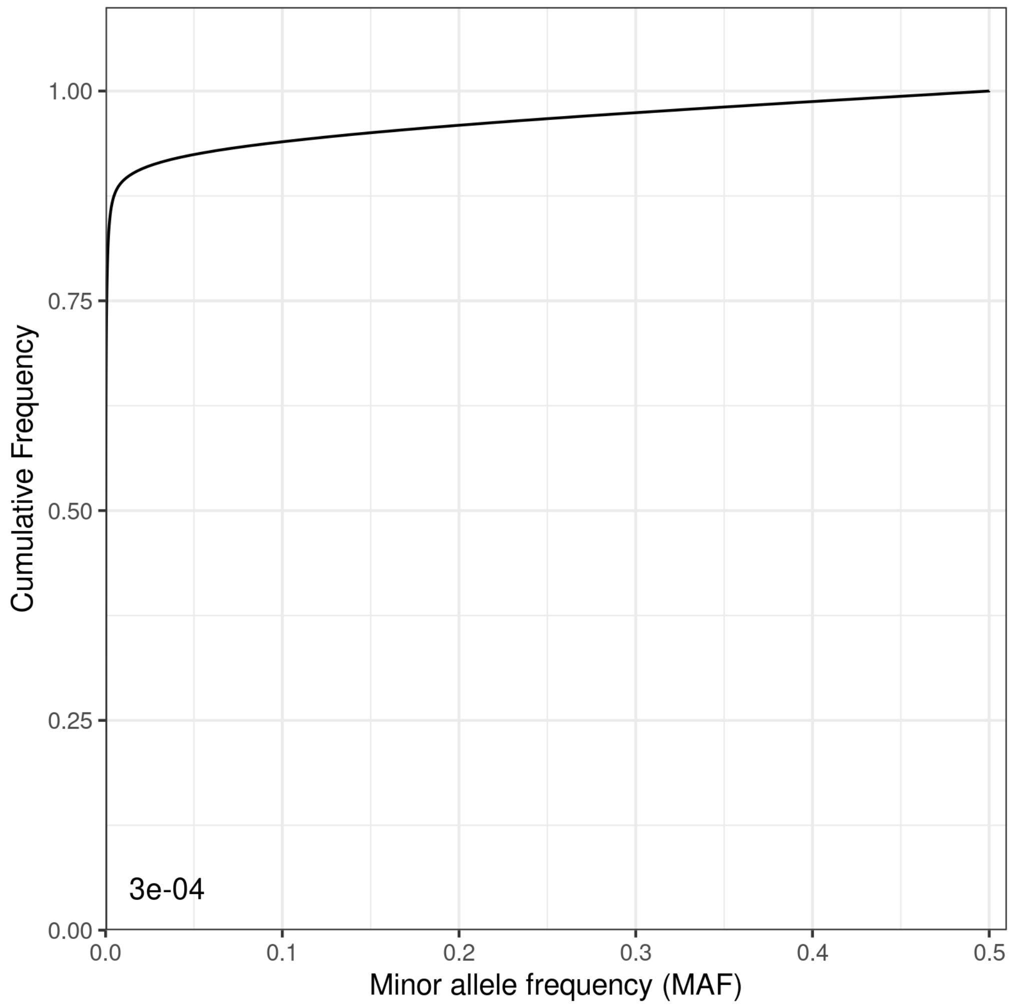

Minor allele frequencies (MAF) across all variants
A cumulative frequency distribution of minor allele frequencies (MAFs) across all autosomal chromosome variants.

INFO score

Following imputation, IMPUTE2 reports an information metric (INFO score) per SNP, which was provided by UKB. The INFO score is a value between 0 and 1, where values near 1 indicate that a SNP has been imputed with high certainty, and 0 indicates a SNP has not been imputed with any certainty. Filtering SNPs by INFO score can be used to exclude poorly imputed SNPs from the analysis. INFO score relates to MAF, as imputation accuracy is lower in low minor allele frequency regions, which can be seen in the below plots. There is no universal cut-off value for imputation, but a cut-off of 0.7 was applied here to balance the quantity of lost data with data quality [1]. This resulted in 53,977,153 SNPs excluded from downstream analysis.

The majority of variants with a low INFO score also had a MAF below 0.01. Following SNP QC, 83,355,424 variants were excluded from the downstream analysis. This left a total of 9,740,199 variants for inclusion in the GWAS. Of these 9,740,199 variants included in the GWAS analysis, 643,442 SNPs were genotyped and the remainder were imputed.

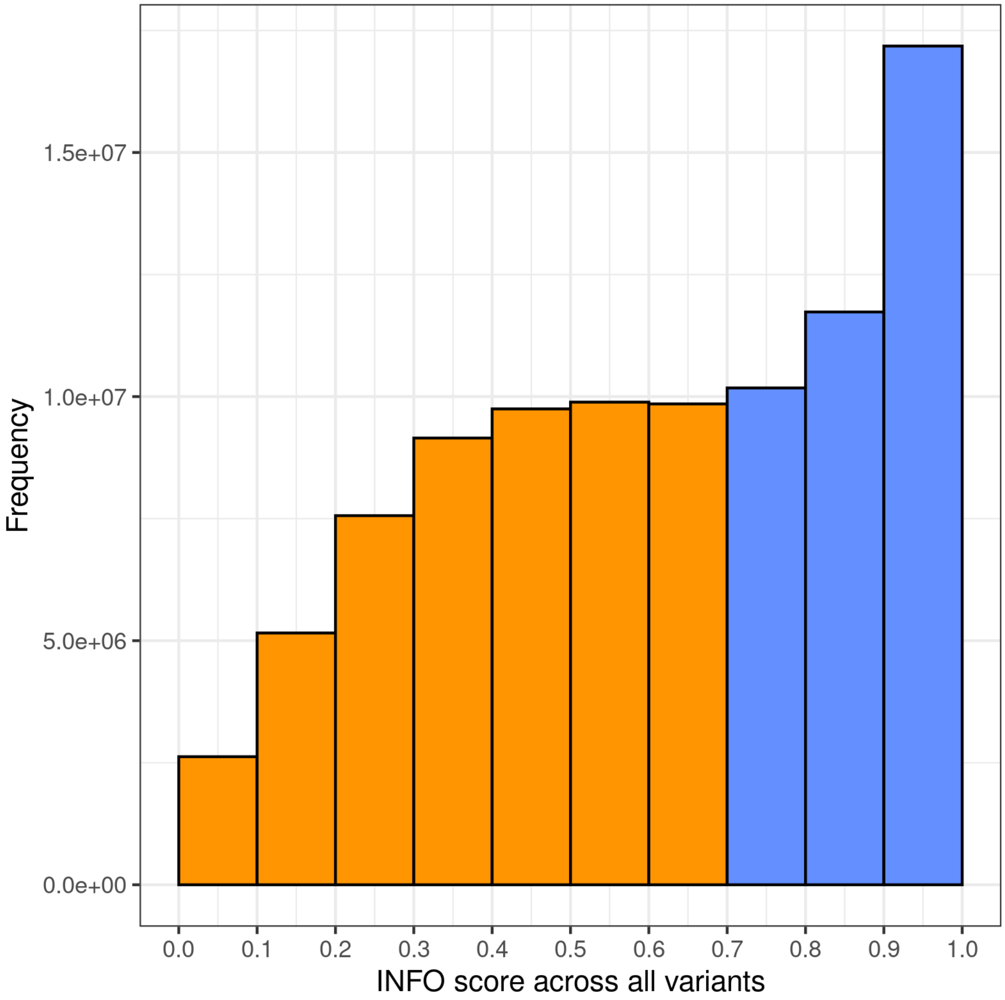

INFO score across all variants

INFO scores across all variants (n = 93,095,623), with variants falling below the INFO score cut-off threshold colored in orange (n = 53,977,153).

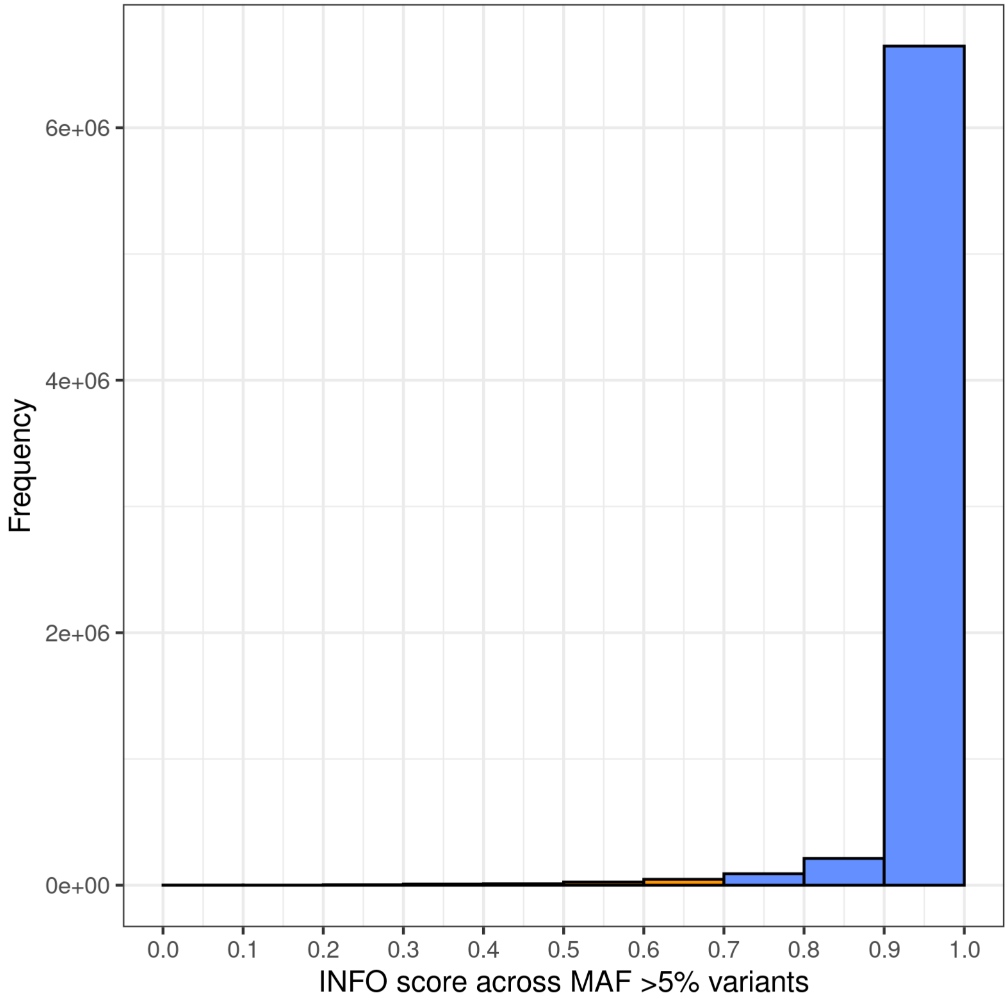

INFO score across common variants
INFO scores across common variants (those with MAF greater than or equal to 5%, n = 7,046,432). Variants falling below the INFO score cut-off threshold are colored in orange (n = 96,722 variants excluded from within this group, 1.3% of the total number of common variants).

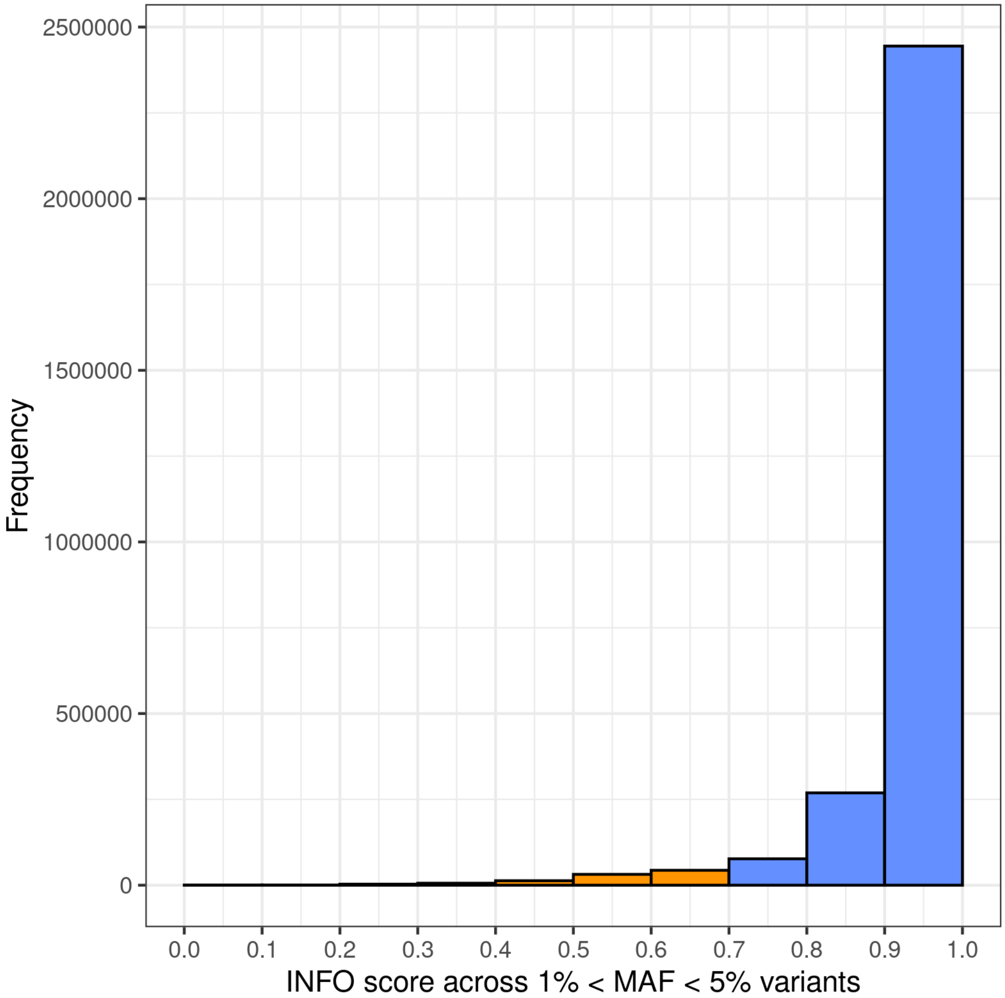

INFO score across low frequency variants

INFO scores across low frequency variants (those with MAF greater than or equal to 1% but less than 5%, n = 2,888,274). Variants falling below the INFO score cut-off threshold are colored in orange (n = 97,785 variants excluded from within this group, 3.3% of the total number of low frequency variants).

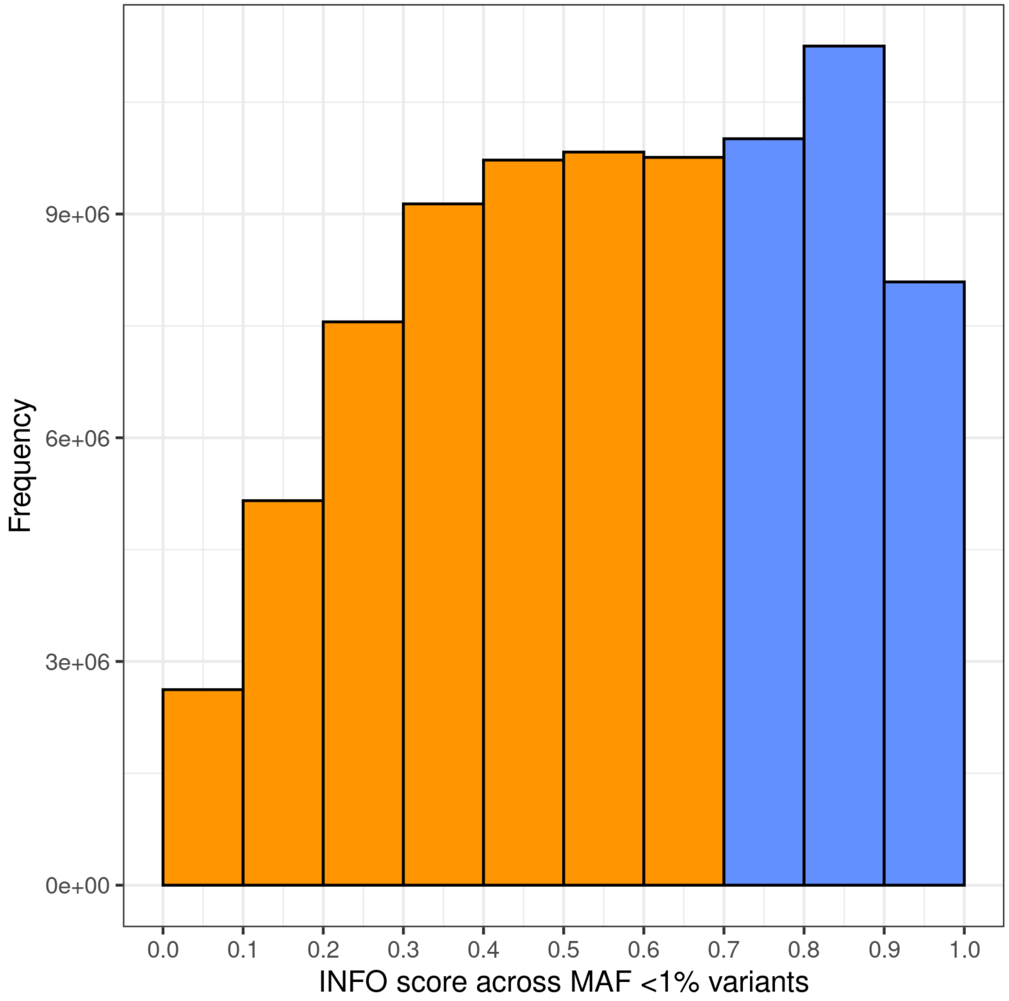

INFO score across rare variants
INFO scores across rare variants (those with MAF less than 1%, n = 83,160,917). Variants falling below the INFO score cut-off threshold are colored in orange (n = 53,782,646 variants excluded from within this group, 64.7% of the total number of rare variants).

References

1. Verma SS, de Andrade M, Tromp G, Kuivaniemi H, Pugh E, Namjou-Khales B, et al. Imputation and quality control steps for combining multiple genome-wide datasets. Front Genet. 2014;5:370. Epub 20141211. doi: 10.3389/fgene.2014.00370. PubMed PMID: 25566314; PubMed Central PMCID: PMCPMC4263197.
