# Supplementary material for: Genetics of vegetarianism: A genome-wide association study
Source: PLoS One. 2023 Oct 4;18(10):e0291305. doi: 10.1371/journal.pone.0291305 (PMC10550162; doi:10.1371/journal.pone.0291305)
Supplement: S3 Appendix — (PDF) [file pone.0291305.s003.pdf]

Phenotype processing methods

In order to perform a GWAS, the quality-controlled data set was split into vegetarianism cases and controls. Any individual who did not pass either the case or control criteria was listed as missing (NA) in the final phenotype file and was therefore not included in the GWAS.

Vegetarianism cases

Dietary phenotype data were collected using two questionnaires: The touchscreen questionnaire, which had ~500K respondents, and the diet by 24-hour recall questionnaire, which captured ~110K of the respondents. The touchscreen questionnaire and the diet by 24-hour recall questionnaire were not mutually exclusive, so both questionnaires were screened separately.

Initial exclusions

Initially, individuals who answered "Do not know" or "Prefer not to answer" from the following data fields were excluded:

- 1329 - Oily fish intake
- 1339 - Non-oily fish intake
- 1349 - Processed meat intake
- 1359 - Poultry intake
- 1369 - Beef intake
- 1379 - Lamb/mutton intake
- 1389 - Pork intake

Each data field had multiple instance columns, where the same question was asked of the individual over multiple time points. An individual was excluded if they responded "Do not know" or "Prefer not to answer" to any instance. In addition, the time since an individual last ate meat was also assessed. The age when last ate meat (3680) instance 0 was subtracted from the age when attended assessment center (21003) instance 0, resulting in a value in whole years for when the individual last ate meat. Individuals with a value of "0" or "1" were excluded (i.e. those individuals who had eaten meat within the previous year). Additionally, those who responded with "Prefer not to answer" were also excluded. Those who had a value of "2" or greater, those who responded with "Do not know" or those who did not answer field 3680 (NA) were retained. The pool of remaining individuals was then screened separately for the touchscreen questionnaire parsing and diet by 24-hour recall questionnaire parsing.

The touchscreen questionnaire screening

The same data fields that were assessed in the initial exclusions were further assessed to include any individual who answered "Never" to all the data fields as cases. Individuals were therefore retained as a case if they had at least one instance within a data field answered as "Never" and every other instance was either "Never" or NA (where the individual had not completed that particular instance) within that data field. Individuals who had responded to any instance with an answer other than "Never" or NA were excluded. Individuals who had not responded to any instance within a data field were also excluded.

The diet by 24-hour recall questionnaire

A number of separate criteria were used to screen the 24-hour recall questionnaire. Data field 20086 (type of special diet followed) was assessed to include any individual who answered

“Vegetarian” or “Vegan”. This data field was recorded at multiple instances. Individuals were therefore filtered to include only those who answered on at least one instance, and that instance was recorded as "Vegetarian" or "Vegan", and any other instance answered was either "Vegetarian", "Vegan" or NA (the individual had not completed that particular instance).
From this pool of individuals who were included as cases, answers to data fields under category 100106 were then assessed. This category included the following data fields (please note the data coding group is shown in brackets for each field):

- 103000 - Meat consumers (100010)
- 103010 - Sausage intake (100016)
- 103020 - Beef intake (100016)
- 103030 - Pork intake (100016)
- 103040 - Lamb intake (100016)
- 103050 - Crumbed or deep-fried poultry intake (100016)
- 103060 - Poultry intake (100016)
- 103070 - Bacon intake (100016)
- 103080 - Ham intake (100016)
- 103090 - Liver intake (100016)
- 103100 - Other meat intake (100016)
- 103130 - Skin removed from poultry (100008)
- 103120 - Fat removed from meat (100008)
- 103140 - Fish consumer (100010)
- 103150 - Tinned tuna intake (100004)
- 103160 - Oily fish intake (100004)
- 103170 - Breaded fish intake (100004)
- 103180 - Battered fish intake (100004)
- 103190 - White fish intake (100004)
- 103200 - Prawns intake (100004)
- 103210 - Lobster/crab intake (100004)
- 103220 - Shellfish intake (100004)
- 103230 - Other fish intake (100004)

Separate exclusion criteria were applied to each separate coding group:

- Fields coded with coding group 100010: individuals who answered "Yes" were excluded
- Fields coded with coding group 100016: exclude all who answered (i.e., only retain those individuals who were NA (did not respond))
- Fields coded with coding group 100008: exclude those who answered "Yes", "No", "Varied" or "Do not know" (i.e. only retain those individuals who responded with "N/A" (not applicable) or NA (did not respond))
- Fields coded with coding group 100004: exclude all who answered (i.e., only retain those individuals who were NA (did not respond))

From this pool of individuals who were included as cases, answers to the following data fields were then assessed (please note the data coding group is shown in brackets for each field):

- 102750 - Sushi intake (100001)
- 20108 - Ingredients in canned soup (86)
- 20109 - Ingredients in homemade soup (86)
- 20090 - Type of fat/oil used in cooking (80)

Separate exclusion criteria were applied to each separate coding group:

- Fields coded with coding group 100001: exclude all who answered (i.e., only retain those individuals who were NA (did not respond))

- Fields coded with coding group 86: exclude those who answered "Fish" or "Meat"
- Fields coded with coding group 80: exclude those who answered "Lard"

Combining the touchscreen questionnaire and the 24-hour recall questionnaire
Screening the two separate questionnaires resulted in two overlapping pools of individuals classed as vegetarianism cases. While the majority of these two pools overlapped, there were some individuals who failed one questionnaire screening but passed the other. These individuals were excluded from the total pool of cases. Some individuals who passed the touchscreen questionnaire were not included within the 24-hour recall cases, as not everyone who filled out the touchscreen questionnaire also filled out the 24-hour recall questionnaire. These individuals were retained for inclusion within the analysis. This resulted in a final pool of 5,324 cases.

Vegetarianism controls

Initial vegetarianism control screening was similar to the case screening, where individuals who answered "Do not know" or "Prefer not to answer" from the following data fields were excluded:

- 1329 - Oily fish intake
- 1339 - Non-oily fish intake
- 1349 - Processed meat intake
- 1359 - Poultry intake
- 1369 - Beef intake
- 1379 - Lamb/mutton intake
- 1389 - Pork intake

Each data field had multiple instance columns, where the same question was asked of the individual over multiple time points. An individual was excluded if they responded "Do not know" or "Prefer not to answer" to any instance.

Remaining individuals were then screened so any individual who answered "Less than once a week", "Once a week", "2-4 times a week", "5-6 times a week" or "Once or more daily" on at least one occasion to any of the above data fields was retained for inclusion as a control. This resulted in a final pool of 329,455 controls.
