# Supplementary material for: Genetics of vegetarianism: A genome-wide association study
Source: PLoS One. 2023 Oct 4;18(10):e0291305. doi: 10.1371/journal.pone.0291305 (PMC10550162; doi:10.1371/journal.pone.0291305)
Supplement: S4 Appendix — (PDF) [file pone.0291305.s004.pdf]

GWAS methods

SAIGE overview

The GWAS in this report was performed using Scalable and Accurate Implementation of GEneralized mixed model (SAIGE), an R package for genome-wide association tests in large-scale data sets and biobanks. SAIGE works with both quantitative and binary traits. It accounts for sample relatedness based on generalized mixed models and allows for model fitting with a full genetic relationship matrix (GRM). It is particularly useful for UKB data, which typically has a far greater number of controls than cases, as it handles case-control imbalance well. GWAS analysis programs which do not cope with uneven numbers of cases and controls produce test statistics which are miscalibrated for unbalanced case-control traits, resulting in false positive associations at rare SNPs. However, SAIGE uses a saddlepoint approximation method (SPA) which has been shown to control for inflated type I error rates, which are often seen in binary traits with unbalanced case-control ratios in related samples. This method has been tested specifically on UKB data [1]. A detailed description of how the program is run can be found within the SAIGE github.

Excluding QC sample and SNP fails

The genotype data were used as input for the first stage of the SAIGE analysis. Samples that failed the QC were marked as missing (NA) within the phenotype file provided to SAIGE within this stage. This ensured that the GWAS was only carried out on individuals that passed the sample QC. Genotyped SNPs with a MAF less than 0.01 were not included within the first stage of the SAIGE analysis.

The imputed data were used as input for the second (final) stage of the SAIGE analysis. Samples that were excluded from the first stage of the analysis were automatically not included in the second stage of analysis. The second stage of analysis was only performed on the SNPs that passed the SNP QC. Following SNP QC, 83,355,424 variants were excluded from the downstream analysis. This left a total of 9,740,199 variants for inclusion in the GWAS.

Step 1: Fitting the null logistic mixed model

In this first stage, the null logistic mixed model was fitted to the genotype data to estimate the variance component and other model parameters.

Prior to fitting the null logistic mixed model, the chromosome-separated post-QC filtered PLINK genotype files were merged using PLINK v1.9. This resulted in one set of PLINK genotype files containing all chromosomes. An accompanying phenotype file was created containing all samples, however the samples that failed the sample QC were marked as missing (NA) and were therefore not included in the analysis by SAIGE. The phenotype file contained the case control status for the individuals of interest, along with a number of key covariates:

- Genetic sex (data field 22001)
- Age when attended the assessment center (data field 21003)
- Assessment center attended for the initial assessment visit (data field 54)
- Genotyping array (listed within the PLINK .fam files)
- Genotyping batch (listed within the PLINK .fam files)
- Principal components 1 to 20 (data field 22009)

Therefore, the input files for this stage were as follows:

- Merged genotype files (in PLINK format)

- A phenotype file containing the case / control classifications and covariates for the GWAS, with samples marked as missing (NA) for all data points if they failed the sample QC.

The input data were used to construct the full genetic relationship matrix (GRM) and estimate the variance ratio for the second stage of the analysis.

Step 2: Performing single-variant association tests
Single-variant association tests were performed between genetic variants and the phenotype by applying SPA to the score test statistics.

The input files for this stage were as follows:
- Imputed BGEN files for each chromosome and their associated BGI files and sample ID order file
- The GMMAT model file and variance ratio files produced by Step 1

In addition to the input data supplied, the following model parameters were selected:
- AlleleOrder: ref-first
- minMAF: 0.01
- minMAC: 20
- is_Firth_beta: TRUE
- pCutoffforFirth: 0.1
- LOCO: TRUE

For binary traits, effect sizes can be estimated more accurately through Firth's bias-reduced logistic regression, so this was applied at this stage using is_Firth_beta = TRUE in the input parameters [2].

Association results produced from this stage are in regard to allele 2.

References

1. Zhou W, Nielsen JB, Fritsche LG, Dey R, Gabrielsen ME, Wolford BN, et al. Efficiently controlling for case-control imbalance and sample relatedness in large-scale genetic association studies. Nature Genetics. 2018;50(9):1335-41. doi: 10.1038/s41588-018-0184-y.

2. Wang X. Firth logistic regression for rare variant association tests. Front Genet. 2014;5:187. Epub 20140619. doi: 10.3389/fgene.2014.00187. PubMed PMID: 24995013; PubMed Central PMCID: PMCPMC4063169.
