# Supplementary material for: Genetics of vegetarianism: A genome-wide association study
Source: PLoS One. 2023 Oct 4;18(10):e0291305. doi: 10.1371/journal.pone.0291305 (PMC10550162; doi:10.1371/journal.pone.0291305)
Supplement: S5 Appendix — (PDF) [file pone.0291305.s005.pdf]

## Genes with Possible Roles in Vegetarianism

***RIOK3*** (RIO Kinase 3) encodes a serine/threonine-protein kinase localized mainly in the cytoplasm [1, 2]. RIOK3 is involved in several biological functions, including ribosome biogenesis, regulation of type I interferon (IFN)-dependent immune responses, chromatin condensation in erythropoiesis, cancer cell proliferation, invasion and metastasis, and psychiatric disorders [2-11].

***NPC1*** (NPC Intracellular Cholesterol Transporter 1) encodes a large protein that resides in the membrane of endosomes and lysosomes and mediates intracellular trafficking of cholesterol and glycolipids [12-20]. Recent data indicate that activation of Rab7 by a trimeric GEF complex that includes RMC1 (C18orf8) is required for lysosomal NPC1-dependent cholesterol export [21]. Mutations in *NPC1* are responsible for 95% of cases of Niemann-Pick disease type C, a lysosomal storage disease characterized by intracellular accumulation of cholesterol and glycosphingolipids in various tissues, with progressive neurological disease being the most significant clinical manifestation [22, 23].

***RMC1*** (Regulator of MON1-CCZ1, previously known as C18orf8) encodes a member of the CCZ1-MON1 complex that functions as a guanine exchange factor (GEF) for Rab7 [21, 24]. It is associated with lysosomes and endosomes [24-26]. RMC1 is required for the stability and function of this complex and is thus necessary for efficient Rab7 function and localization to endosomes, endosomal/autophagic flux, cellular LDL-cholesterol uptake, and NPC1-dependent lysosomal cholesterol export [21, 24]. Interestingly, the expression of *RMC1* and *NPC1* (see below) is coregulated in human tissues [27]. *RMC1*-deficient cells exhibit severe defects in Rab7 activation and LDL trafficking, with swelling of the late endosomal/lysosomal compartment and marked lysosomal cholesterol accumulation [21].

***TMEM241*** (Transmembrane Protein 241) encodes a multi-pass transmembrane protein thought to be localized to the Golgi apparatus. It is homologous to the yeast VRG4 GDP-mannose transporter that is involved in glycoprotein modification and mannosylation of sphingolipids [28]. TMEM241 is therefore believed to be a nucleotide sugar transporter with a role in lipid metabolism, a notion that is supported by the finding that decreased expression of *TMEM241* is associated with increased serum triglyceride levels in a Mexican population [29]. *TMEM241* is the most overexpressed gene in transient myeloproliferative disorder and acute megakaryoblastic leukemia, compared to normal megakaryocytes, suggesting a role in leukemic transformation in the megakaryocytic lineage [30].

***VRK2*** (Vaccinia-related Kinase 2) encodes a serine/threonine kinase that can localize to the cytoplasm and nucleus in soluble form or to the endoplasmic reticulum and mitochondrial membranes, depending on the isoform [31]. It plays multiple roles in various signaling pathways, tumor biology, and viral infection [31-43]. In addition, there is evidence that VRK2 plays a role in neurodevelopment and the pathogenesis of neurologic and psychiatric disorders [44-50].

***TMEM132D*** (Transmembrane Protein 132D) encodes a member of a family of transmembrane proteins expressed in the central nervous system that are thought to act as adhesion molecules connecting the extracellular matrix with the actin cytoskeleton [51]. *TMEM132D* is believed to be expressed in mature oligodendrocytes [52]. Members of the *TMEM132* family show differential expression during mouse nervous system development and the *C. elegans* *TMEM132* ortholog plays a role in neuronal morphogenesis [53, 54]. There is strong evidence for a role of *TMEM132D* in panic disorder and anxiety; GWAS studies have shown significant

association of *TMEM132D*-associated variants with anxiety and panic disorder and data in mice and humans indicate that anxiety and panic disorder are associated with increased expression of TMEM132D RNA [55-63]. In addition, *TMEM132D* may play a role in ovarian cancer, small cell lung cancer, and colorectal cancer [64-66].

***METAP2*** (Methionyl Aminopeptidase 2) encodes a member of the methionyl aminopeptidase family with two major functions in protein synthesis: It catalyzes the removal of the initiator methionine residue from nascent proteins, which is a prerequisite for most protein processing events, and it binds to the eukaryotic initiation factor 2-□ (eIF2□) thereby protecting it from inhibitory phosphorylation and promoting protein synthesis [67]. METAP2 inhibitors have shown activity against cancer, obesity, and autoimmunity in preclinical and clinical studies by mechanisms that are not well understood [67].

***USP44*** (Ubiquitin Specific Peptidase 44) encodes a deubiquitinating enzyme that deubiquitinates CDC20 and plays an important role in regulating the spindle checkpoint in mitosis [68, 69]. It also regulates stem cell differentiation and autophagy through modulating the ubiquitylation of H2B [70, 71]. USP44 acts as a tumor suppressor in some neoplasms [69, 72-78], but it correlates with tumorigenesis and tumor progression in others [79-82]. Mutation of *USP44* has been linked to autosomal recessive intellectual disability in rare individuals from consanguineous families [83, 84].

***CDYL2*** (Chromodomain Y Like 2) encodes a chromodomain-containing protein that binds to methylated lysine residues within ARK(S/T) motifs in histones including H3K9me3, H3tK27me3, and H1.4K26me3 [85]. It is overexpressed in breast cancer where it is involved in epigenetic transcriptional regulation and associated with poor prognosis [86]. There is evidence that different transcript variants of CDYL2 play distinct roles in breast cancer biology [87]. Downregulation of CDYL2 in hepatocellular carcinoma is associated with poor survival [88].

***ZNF407*** (Zinc Finger Protein 407) encodes a zinc finger protein that regulates insulin-stimulated glucose uptake in adipocytes by regulating Glut4 mRNA transcription and splicing [89]. Overexpression of ZNF407 in transgenic mice enhanced the transcription of PPAR target genes, including GLUT4, in muscle and resulted in improved glucose tolerance and decreased weight [90]. The mechanisms by which ZNF407 regulates transcriptional activation by PPARs are not clear. Mutations and chromosomal translocation involving the ZNF407 gene are linked to intellectual disability and autism [91, 92].

***CDH4*** (Cadherin 4) encodes a member of the classical subfamily of cadherins, transmembrane proteins that mediate calcium-dependent cell-cell adhesion [93]. It forms both homodimers and heterodimers with CDH2 and plays an important role in neurodevelopment [94-98]. CDH4 also plays a role in several human tumors. It appears to have tumor suppressor role in gastrointestinal cancers, nasopharyngeal carcinoma, salivary adenoid cystic carcinoma, and lung cancer [99-102]. On the other hand, CDH4 promotes tumorigenesis, invasion, and/or metastasis in osteosarcoma, glioblastoma, and bladder cancer [103-105].

## References

1. Anaya P, Evans SC, Dai C, Lozano G, May GS. Isolation of the Aspergillus nidulans sudD gene and its human homologue. Gene. 1998;211(2):323-9. doi: 10.1016/s0378-1119(98)00115-2. PubMed PMID: 9602165.

2. Baumas K, Soudet J, Caizergues-Ferrer M, Faubladier M, Henry Y, Mougin A. Human RioK3 is a novel component of cytoplasmic pre-40S pre-ribosomal particles. RNA Biol. 2012;9(2):162-74. Epub 20120201. doi: 10.4161/rna.18810. PubMed PMID: 22418843; PubMed Central PMCID: PMCPMC3346313.

3. Feng J, De Jesus PD, Su V, Han S, Gong D, Wu NC, et al. RIOK3 is an adaptor protein required for IRF3-mediated antiviral type I interferon production. J Virol. 2014;88(14):7987-97. Epub 20140507. doi: 10.1128/JVI.00643-14. PubMed PMID: 24807708; PubMed Central PMCID: PMCPMC4097797.

4. Zhang L, Flygare J, Wong P, Lim B, Lodish HF. miR-191 regulates mouse erythroblast enucleation by down-regulating Riok3 and Mxi1. Genes Dev. 2011;25(2):119-24. Epub 20101231. doi: 10.1101/gad.1998711. PubMed PMID: 21196494; PubMed Central PMCID: PMCPMC3022257.

5. Kimmelman AC, Hezel AF, Aguirre AJ, Zheng H, Paik JH, Ying H, et al. Genomic alterations link Rho family of GTPases to the highly invasive phenotype of pancreas cancer. Proc Natl Acad Sci U S A. 2008;105(49):19372-7. Epub 20081202. doi: 10.1073/pnas.0809966105. PubMed PMID: 19050074; PubMed Central PMCID: PMCPMC2614768.

6. Singleton DC, Rouhi P, Zois CE, Haider S, Li JL, Kessler BM, et al. Hypoxic regulation of RIOK3 is a major mechanism for cancer cell invasion and metastasis. Oncogene. 2015;34(36):4713-22. Epub 20141208. doi: 10.1038/onc.2014.396. PubMed PMID: 25486436; PubMed Central PMCID: PMCPMC4430306.

7. Guo L, Ni Z, Wei G, Cheng W, Huang X, Yue W. Epigenome-wide DNA methylation analysis of whole blood cells derived from patients with GAD and OCD in the Chinese Han population. Transl Psychiatry. 2022;12(1):465. Epub 20221107. doi: 10.1038/s41398-022-02236-x. PubMed PMID: 36344488; PubMed Central PMCID: PMCPMC9640561.

8. Qin H, Sun R, Guo X, Fang L, Xu M, Teng Y, et al. RIOK3 promotes mTORC1 activation by facilitating SLC7A2-mediated arginine uptake in pancreatic ductal adenocarcinoma. Aging (Albany NY). 2023;15(4):1039-51. Epub 20230224. doi: 10.18632/aging.204528. PubMed PMID: 36880835; PubMed Central PMCID: PMCPMC10008507.

9. Bisom TC, White LA, Lanchy JM, Lodmell JS. RIOK3 and Its Alternatively Spliced Isoform Have Disparate Roles in the Innate Immune Response to Rift Valley Fever Virus (MP12) Infection. Viruses. 2022;14(9). Epub 20220917. doi: 10.3390/v14092064. PubMed PMID: 36146870; PubMed Central PMCID: PMCPMC9502082.

10. Shen Y, Tang K, Chen D, Hong M, Sun F, Wang S, et al. Riok3 inhibits the antiviral immune response by facilitating TRIM40-mediated RIG-I and MDA5 degradation. Cell Rep. 2021;35(12):109272. doi: 10.1016/j.celrep.2021.109272. PubMed PMID: 34161773; PubMed Central PMCID: PMCPMC8363743.

11. Zhang T, Ji D, Wang P, Liang D, Jin L, Shi H, et al. The atypical protein kinase RIOK3 contributes to glioma cell proliferation/survival, migration/invasion and the AKT/mTOR signaling pathway. Cancer Lett. 2018;415:151-63. Epub 20171209. doi: 10.1016/j.canlet.2017.12.010. PubMed PMID: 29233656.

12. Watari H, Blanchette-Mackie EJ, Dwyer NK, Glick JM, Patel S, Neufeld EB, et al. Niemann-Pick C1 protein: obligatory roles for N-terminal domains and lysosomal targeting in cholesterol mobilization. Proc Natl Acad Sci U S A. 1999;96(3):805-10. Epub 1999/02/03. doi: 10.1073/pnas.96.3.805. PubMed PMID: 9927649; PubMed Central PMCID: PMCPMC15306.

13. Carstea ED, Morris JA, Coleman KG, Loftus SK, Zhang D, Cummings C, et al. Niemann-Pick C1 disease gene: homology to mediators of cholesterol homeostasis. Science. 1997;277(5323):228-31. doi: 10.1126/science.277.5323.228. PubMed PMID: 9211849.

14. Davies JP, Ioannou YA. Topological analysis of Niemann-Pick C1 protein reveals that the membrane orientation of the putative sterol-sensing domain is identical to those of 3-hydroxy-3-methylglutaryl-CoA reductase and sterol regulatory element binding protein cleavage-activating protein. J Biol Chem. 2000;275(32):24367-74. doi: 10.1074/jbc.M002184200. PubMed PMID: 10821832.

15. Infante RE, Wang ML, Radhakrishnan A, Kwon HJ, Brown MS, Goldstein JL. NPC2 facilitates bidirectional transfer of cholesterol between NPC1 and lipid bilayers, a step in cholesterol egress from lysosomes. Proc Natl Acad Sci U S A. 2008;105(40):15287-92. Epub 20080904. doi: 10.1073/pnas.0807328105. PubMed PMID: 18772377; PubMed Central PMCID: PMCPMC2563079.

16. Gong X, Qian H, Zhou X, Wu J, Wan T, Cao P, et al. Structural Insights into the Niemann-Pick C1 (NPC1)-Mediated Cholesterol Transfer and Ebola Infection. Cell. 2016;165(6):1467-78. Epub 20160526. doi: 10.1016/j.cell.2016.05.022. PubMed PMID: 27238017; PubMed Central PMCID: PMCPMC7111323.

17. Blom TS, Linder MD, Snow K, Pihko H, Hess MW, Jokitalo E, et al. Defective endocytic trafficking of NPC1 and NPC2 underlying infantile Niemann-Pick type C disease. Hum Mol Genet. 2003;12(3):257-72. doi: 10.1093/hmg/ddg025. PubMed PMID: 12554680.

18. Kwon HJ, Abi-Mosleh L, Wang ML, Deisenhofer J, Goldstein JL, Brown MS, et al. Structure of N-terminal domain of NPC1 reveals distinct subdomains for binding and transfer of cholesterol. Cell. 2009;137(7):1213-24. doi: 10.1016/j.cell.2009.03.049. PubMed PMID: 19563754; PubMed Central PMCID: PMCPMC2739658.

19. Li X, Lu F, Trinh MN, Schmiege P, Seemann J, Wang J, et al. 3.3 A structure of Niemann-Pick C1 protein reveals insights into the function of the C-terminal luminal domain in cholesterol transport. Proc Natl Acad Sci U S A. 2017;114(34):9116-21. Epub 20170807. doi: 10.1073/pnas.1711716114. PubMed PMID: 28784760; PubMed Central PMCID: PMCPMC5576846.

20. Zhang M, Dwyer NK, Neufeld EB, Love DC, Cooney A, Comly M, et al. Sterol-modulated glycolipid sorting occurs in niemann-pick C1 late endosomes. J Biol Chem. 2001;276(5):3417-25. Epub 20001013. doi: 10.1074/jbc.M005393200. PubMed PMID: 11032830.

21. van den Boomen DJH, Sienkiewicz A, Berlin I, Jongsma MLM, van Elsland DM, Luzio JP, et al. A trimeric Rab7 GEF controls NPC1-dependent lysosomal cholesterol export. Nat Commun. 2020;11(1):5559. Epub 20201103. doi: 10.1038/s41467-020-19032-0. PubMed PMID: 33144569; PubMed Central PMCID: PMCPMC7642327.

22. Patterson MC. A riddle wrapped in a mystery: understanding Niemann-Pick disease, type C. Neurologist. 2003;9(6):301-10. doi: 10.1097/01.nrl.0000094627.78754.5b. PubMed PMID: 14629784.

23. Vanier MT. Niemann-Pick diseases. Handb Clin Neurol. 2013;113:1717-21. doi: 10.1016/b978-0-444-59565-2.00041-1. PubMed PMID: 23622394.

24. Vaites LP, Paulo JA, Huttlin EL, Harper JW. Systematic Analysis of Human Cells Lacking ATG8 Proteins Uncovers Roles for GABARAPs and the CCZ1/MON1 Regulator C18orf8/RMC1 in Macroautophagic and Selective Autophagic Flux. Mol Cell Biol. 2018;38(1). Epub 2017/10/19. doi: 10.1128/mcb.00392-17. PubMed PMID: 29038162; PubMed Central PMCID: PMCPMC5730722.

25. Schröder B, Wrocklage C, Pan C, Jäger R, Kösters B, Schäfer H, et al. Integral and associated lysosomal membrane proteins. Traffic. 2007;8(12):1676-86. Epub 20070926. doi: 10.1111/j.1600-0854.2007.00643.x. PubMed PMID: 17897319.

26. Itzhak DN, Tyanova S, Cox J, Borner GH. Global, quantitative and dynamic mapping of protein subcellular localization. Elife. 2016;5. Epub 20160609. doi: 10.7554/eLife.16950. PubMed PMID: 27278775; PubMed Central PMCID: PMCPMC4959882.

27. Zhao W, Zhang Q, Wang J, Yu H, Zhen X, Li L, et al. Novel Indel Variation of NPC1 Gene Associates With Risk of Sudden Cardiac Death. Front Genet. 2022;13:869859. Epub 20220411. doi: 10.3389/fgene.2022.869859. PubMed PMID: 35480314; PubMed Central PMCID: PMCPMC9035640.

28. Dean N, Zhang YB, Poster JB. The VRG4 gene is required for GDP-mannose transport into the lumen of the Golgi in the yeast, Saccharomyces cerevisiae. J Biol Chem. 1997;272(50):31908-14. doi: 10.1074/jbc.272.50.31908. PubMed PMID: 9395539.

29. Rodríguez A, Gonzalez L, Ko A, Alvarez M, Miao Z, Bhagat Y, et al. Molecular Characterization of the Lipid Genome-Wide Association Study Signal on Chromosome 18q11.2 Implicates HNF4A-Mediated Regulation of the TMEM241 Gene. Arterioscler Thromb Vasc Biol. 2016;36(7):1350-5. Epub 2016/05/21. doi: 10.1161/atvbaha.116.307182. PubMed PMID: 27199446; PubMed Central PMCID: PMCPMC5154300.

30. Pelleri MC, Piovesan A, Caracausi M, Berardi AC, Vitale L, Strippoli P. Integrated differential transcriptome maps of Acute Megakaryoblastic Leukemia (AMKL) in children with or without Down Syndrome (DS). BMC Med Genomics. 2014;7:63. Epub 20141205. doi: 10.1186/s12920-014-0063-z. PubMed PMID: 25476127; PubMed Central PMCID: PMCPMC4304173.

31. Blanco S, Klimcakova L, Vega FM, Lazo PA. The subcellular localization of vaccinia-related kinase-2 (VRK2) isoforms determines their different effect on p53 stability in tumour cell lines. Febs j. 2006;273(11):2487-504. doi: 10.1111/j.1742-4658.2006.05256.x. PubMed PMID: 16704422.

32. Fernández IF, Blanco S, Lozano J, Lazo PA. VRK2 inhibits mitogen-activated protein kinase signaling and inversely correlates with ErbB2 in human breast cancer. Mol Cell Biol. 2010;30(19):4687-97. Epub 20100802. doi: 10.1128/mcb.01581-09. PubMed PMID: 20679487; PubMed Central PMCID: PMCPMC2950518.

33. Blanco S, Sanz-García M, Santos CR, Lazo PA. Modulation of interleukin-1 transcriptional response by the interaction between VRK2 and the JIP1 scaffold protein. PLoS One. 2008;3(2):e1660. Epub 20080220. doi: 10.1371/journal.pone.0001660. PubMed PMID: 18286207; PubMed Central PMCID: PMCPMC2243017.

34. Blanco S, Santos C, Lazo PA. Vaccinia-related kinase 2 modulates the stress response to hypoxia mediated by TAK1. Mol Cell Biol. 2007;27(20):7273-83. Epub 20070820. doi: 10.1128/mcb.00025-07. PubMed PMID: 17709393; PubMed Central PMCID: PMCPMC2168905.

35. Nichols RJ, Wiebe MS, Traktman P. The vaccinia-related kinases phosphorylate the N' terminus of BAF, regulating its interaction with DNA and its retention in the nucleus. Mol Biol Cell. 2006;17(5):2451-64. Epub 20060222. doi: 10.1091/mbc.e05-12-1179. PubMed PMID: 16495336; PubMed Central PMCID: PMCPMC1446082.

36. Zhu H, Li Q, Zhao Y, Peng H, Guo L, Zhu J, et al. Vaccinia-related kinase 2 drives pancreatic cancer progression by protecting Plk1 from Chfr-mediated degradation. Oncogene. 2021;40(28):4663-74. Epub 20210617. doi: 10.1038/s41388-021-01893-4. PubMed PMID: 34140642.

37. Peled M, Adam K, Mor A. Data on the identification of VRK2 as a mediator of PD-1 function. Data Brief. 2021;37:107168. Epub 20210521. doi: 10.1016/j.dib.2021.107168. PubMed PMID: 34113705; PubMed Central PMCID: PMCPMC8170101.

38. Chen S, Du Y, Xu B, Li Q, Yang L, Jiang Z, et al. Vaccinia-related kinase 2 blunts sorafenib's efficacy against hepatocellular carcinoma by disturbing the apoptosis-autophagy balance. Oncogene. 2021;40(19):3378-93. Epub 20210419. doi: 10.1038/s41388-021-01780-y. PubMed PMID: 33875785.

39. He WR, Cao LB, Yang YL, Hua D, Hu MM, Shu HB. VRK2 is involved in the innate antiviral response by promoting mitostress-induced mtDNA release. Cell Mol Immunol. 2021;18(5):1186-96. Epub 20210330. doi: 10.1038/s41423-021-00673-0. PubMed PMID: 33785841; PubMed Central PMCID: PMCPMC8093274.

40. Yin Y, Xie CM, Li H, Tan M, Chen G, Schiff R, et al. The FBXW2-MSX2-SOX2 axis regulates stem cell property and drug resistance of cancer cells. Proc Natl Acad Sci U S A. 2019;116(41):20528-38. Epub 20190923. doi: 10.1073/pnas.1905973116. PubMed PMID: 31548378; PubMed Central PMCID: PMCPMC6789910.

41. Rico AB, Wang Z, Olson AT, Linville AC, Bullard BL, Weaver EA, et al. The Vaccinia Virus (VACV) B1 and Cellular VRK2 Kinases Promote VACV Replication Factory Formation through Phosphorylation-Dependent Inhibition of VACV B12. J Virol. 2019;93(20). Epub 20190930. doi: 10.1128/jvi.00855-19. PubMed PMID: 31341052; PubMed Central PMCID: PMCPMC6798115.

42. Vázquez-Cedeira M, Lazo PA. Human VRK2 (Vaccinia-related Kinase 2) Modulates Tumor Cell Invasion by Hyperactivation of NFAT1 and Expression of Cyclooxygenase-2*. Journal of Biological Chemistry. 2012;287(51):42739-50. doi: https://doi.org/10.1074/jbc.M112.404285.

43. Hirata N, Suizu F, Matsuda-Lennikov M, Tanaka T, Edamura T, Ishigaki S, et al. Functional characterization of lysosomal interaction of Akt with VRK2. Oncogene. 2018;37(40):5367-86. Epub 20180605. doi: 10.1038/s41388-018-0330-0. PubMed PMID: 29872222; PubMed Central PMCID: PMCPMC6172193.

44. Kim S, Lee D, Lee J, Song H, Kim HJ, Kim KT. Vaccinia-Related Kinase 2 Controls the Stability of the Eukaryotic Chaperonin TRiC/CCT by Inhibiting the Deubiquitinating Enzyme USP25. Mol Cell Biol. 2015;35(10):1754-62. Epub 20150309. doi: 10.1128/mcb.01325-14. PubMed PMID: 25755282; PubMed Central PMCID: PMCPMC4405641.

45. Lee J, Lee S, Ryu YJ, Lee D, Kim S, Seo JY, et al. Vaccinia-related kinase 2 plays a critical role in microglia-mediated synapse elimination during neurodevelopment. Glia. 2019;67(9):1667-79. Epub 20190503. doi: 10.1002/glia.23638. PubMed PMID: 31050055.

46. Li M, Yue W. VRK2, a Candidate Gene for Psychiatric and Neurological Disorders. Mol Neuropsychiatry. 2018;4(3):119-33. Epub 20181107. doi: 10.1159/000493941. PubMed PMID: 30643786; PubMed Central PMCID: PMCPMC6323383.

47. Liu J, Tong L, Song S, Niu Y, Li J, Wu X, et al. Novel and de novo mutations in pediatric refractory epilepsy. Mol Brain. 2018;11(1):48. Epub 20180905. doi: 10.1186/s13041-018-0392-5. PubMed PMID: 30185235; PubMed Central PMCID: PMCPMC6125990.

48. Jeong YH, Choi JH, Lee D, Kim S, Kim KT. Vaccinia-related kinase 2 modulates role of dysbindin by regulating protein stability. J Neurochem. 2018;147(5):609-25. Epub 20181030. doi: 10.1111/jnc.14562. PubMed PMID: 30062698.

49. Lee E, Ryu HG, Kim S, Lee D, Jeong YH, Kim KT. Glycogen synthase kinase 3β suppresses polyglutamine aggregation by inhibiting Vaccinia-related kinase 2 activity. Sci Rep. 2016;6:29097. Epub 20160705. doi: 10.1038/srep29097. PubMed PMID: 27377031; PubMed Central PMCID: PMCPMC4932512.

50. Tesli M, Wirgenes KV, Hughes T, Bettella F, Athanasiu L, Hoseth ES, et al. VRK2 gene expression in schizophrenia, bipolar disorder and healthy controls. Br J Psychiatry. 2016;209(2):114-20. Epub 20160303. doi: 10.1192/bjp.bp.115.161950. PubMed PMID: 26941264.

51. Sanchez-Pulido L, Ponting CP. TMEM132: an ancient architecture of cohesin and immunoglobulin domains define a new family of neural adhesion molecules. Bioinformatics. 2018;34(5):721-4. doi: 10.1093/bioinformatics/btx689.

52. Nomoto H, Yonezawa T, Itoh K, Ono K, Yamamoto K, Oohashi T, et al. Molecular cloning of a novel transmembrane protein MOLT expressed by mature oligodendrocytes. J Biochem. 2003;134(2):231-8. doi: 10.1093/jb/mvg135. PubMed PMID: 12966072.

53. Wang Y, Herzig G, Molano C, Liu A. Differential expression of the Tmem132 family genes in the developing mouse nervous system. Gene Expr Patterns. 2022;45:119257. Epub 20220608. doi: 10.1016/j.gep.2022.119257. PubMed PMID: 35690356.

54. Wang X, Jiang W, Luo S, Yang X, Wang C, Wang B, et al. The C. elegans homolog of human panic-disorder risk gene TMEM132D orchestrates neuronal morphogenesis through the WAVE-regulatory complex. Mol Brain. 2021;14(1):54. Epub 20210316. doi: 10.1186/s13041-021-00767-w. PubMed PMID: 33726789; PubMed Central PMCID: PMCPMC7962252.

55. Erhardt A, Czibere L, Roeske D, Lucae S, Unschuld PG, Ripke S, et al. TMEM132D, a new candidate for anxiety phenotypes: evidence from human and mouse studies. Mol Psychiatry. 2011;16(6):647-63. Epub 20100406. doi: 10.1038/mp.2010.41. PubMed PMID: 20368705.

56. Quast C, Altmann A, Weber P, Arloth J, Bader D, Heck A, et al. Rare variants in TMEM132D in a case-control sample for panic disorder. Am J Med Genet B Neuropsychiatr Genet. 2012;159b(8):896-907. Epub 20120822. doi: 10.1002/ajmg.b.32096. PubMed PMID: 22911938.

57. Erhardt A, Akula N, Schumacher J, Czamara D, Karbalai N, Müller-Myhsok B, et al. Replication and meta-analysis of TMEM132D gene variants in panic disorder. Transl Psychiatry. 2012;2(9):e156. Epub 20120904. doi: 10.1038/tp.2012.85. PubMed PMID: 22948381; PubMed Central PMCID: PMCPMC3565207.

58. Haaker J, Lonsdorf TB, Raczka KA, Mechias ML, Gartmann N, Kalisch R. Higher anxiety and larger amygdala volumes in carriers of a TMEM132D risk variant for panic disorder. Transl Psychiatry. 2014;4(2):e357. Epub 20140204. doi: 10.1038/tp.2014.1. PubMed PMID: 24495968; PubMed Central PMCID: PMCPMC3944634.

59. Inoue A, Akiyoshi J, Muronaga M, Masuda K, Aizawa S, Hirakawa H, et al. Association of TMEM132D, COMT, and GABRA6 genotypes with cingulate, frontal cortex and hippocampal emotional processing in panic and major depressive disorder. Int J Psychiatry Clin Pract. 2015;19(3):192-200. Epub 20150514. doi: 10.3109/13651501.2015.1043133. PubMed PMID: 25974322.

60. Howe AS, Buttenschøn HN, Bani-Fatemi A, Maron E, Otowa T, Erhardt A, et al. Candidate genes in panic disorder: meta-analyses of 23 common variants in major anxiogenic pathways. Mol Psychiatry. 2016;21(5):665-79. Epub 20150922. doi: 10.1038/mp.2015.138. PubMed PMID: 26390831.

61. Shimada-Sugimoto M, Otowa T, Miyagawa T, Khor SS, Omae Y, Toyo-Oka L, et al. Polymorphisms in the TMEM132D region are associated with panic disorder in HLA-DRB1*13:02-negative individuals of a Japanese population. Hum Genome Var. 2016;3:16001. Epub 20160225. doi: 10.1038/hgv.2016.1. PubMed PMID: 27081567; PubMed Central PMCID: PMCPMC4766370.

62. Hodgson K, Almasy L, Knowles EE, Kent JW, Curran JE, Dyer TD, et al. Genome-wide significant loci for addiction and anxiety. Eur Psychiatry. 2016;36:47-54. Epub 20160616. doi: 10.1016/j.eurpsy.2016.03.004. PubMed PMID: 27318301; PubMed Central PMCID: PMCPMC5483998.

63. Naik RR, Sotnikov SV, Diepold RP, Iurato S, Markt PO, Bultmann A, et al. Polymorphism in Tmem132d regulates expression and anxiety-related behavior through binding of RNA polymerase II complex. Transl Psychiatry. 2018;8(1):1. Epub 20180110. doi: 10.1038/s41398-017-0025-2. PubMed PMID: 29317594; PubMed Central PMCID: PMCPMC5802467.

64. Karapetsas A, Giannakakis A, Dangaj D, Lanitis E, Kynigopoulos S, Lambropoulou M, et al. Overexpression of GPC6 and TMEM132D in Early Stage Ovarian Cancer Correlates with CD8+ T-Lymphocyte Infiltration and Increased Patient Survival. Biomed Res Int. 2015;2015:712438. Epub 20150913. doi: 10.1155/2015/712438. PubMed PMID: 26448945; PubMed Central PMCID: PMCPMC4584051.

65. Iwakawa R, Kohno T, Totoki Y, Shibata T, Tsuchihara K, Mimaki S, et al. Expression and clinical significance of genes frequently mutated in small cell lung cancers defined by whole exome/RNA sequencing. Carcinogenesis. 2015;36(6):616-21. Epub 20150411. doi: 10.1093/carcin/bgv026. PubMed PMID: 25863124; PubMed Central PMCID: PMCPMC4462675.

66. Yu J, Wu WK, Li X, He J, Li XX, Ng SS, et al. Novel recurrently mutated genes and a prognostic mutation signature in colorectal cancer. Gut. 2015;64(4):636-45. Epub 20140620. doi: 10.1136/gutjnl-2013-306620. PubMed PMID: 24951259; PubMed Central PMCID: PMCPMC4392212.

67. Goya Grocin A, Kallemeijn WW, Tate EW. Targeting methionine aminopeptidase 2 in cancer, obesity, and autoimmunity. Trends Pharmacol Sci. 2021;42(10):870-82. Epub 20210823. doi: 10.1016/j.tips.2021.07.004. PubMed PMID: 34446297.

68. Stegmeier F, Rape M, Draviam VM, Nalepa G, Sowa ME, Ang XL, et al. Anaphase initiation is regulated by antagonistic ubiquitination and deubiquitination activities. Nature. 2007;446(7138):876-81. doi: 10.1038/nature05694. PubMed PMID: 17443180.

69. Zhang Y, Foreman O, Wigle DA, Kosari F, Vasmatzis G, Salisbury JL, et al. USP44 regulates centrosome positioning to prevent aneuploidy and suppress tumorigenesis. J Clin Invest. 2012;122(12):4362-74. Epub 20121126. doi: 10.1172/jci63084. PubMed PMID: 23187126; PubMed Central PMCID: PMCPMC3533537.

70. Fuchs G, Shema E, Vesterman R, Kotler E, Wolchinsky Z, Wilder S, et al. RNF20 and USP44 Regulate Stem Cell Differentiation by Modulating H2B Monoubiquitylation. Molecular Cell. 2012;46(5):662-73. doi: https://doi.org/10.1016/j.molcel.2012.05.023.

71. Chen S, Jing Y, Kang X, Yang L, Wang DL, Zhang W, et al. Histone H2B monoubiquitination is a critical epigenetic switch for the regulation of autophagy. Nucleic Acids Res. 2017;45(3):1144-58. doi: 10.1093/nar/gkw1025. PubMed PMID: 28180298; PubMed Central PMCID: PMCPMC5388390.

72. Sloane MA, Wong JW, Perera D, Nunez AC, Pimanda JE, Hawkins NJ, et al. Epigenetic inactivation of the candidate tumor suppressor USP44 is a frequent and early event in colorectal neoplasia. Epigenetics. 2014;9(8):1092-100. Epub 20140516. doi: 10.4161/epi.29222. PubMed PMID: 24837038; PubMed Central PMCID: PMCPMC4164494.

73. Cheng J, Demeulemeester J, Wedge DC, Vollan HKM, Pitt JJ, Russnes HG, et al. Pan-cancer analysis of homozygous deletions in primary tumours uncovers rare tumour suppressors. Nat Commun. 2017;8(1):1221. Epub 20171031. doi: 10.1038/s41467-017-01355-0. PubMed PMID: 29089486; PubMed Central PMCID: PMCPMC5663922.

74. Zhang YK, Tian WZ, Zhang RS, Zhang YJ, Ma HT. Ubiquitin-specific protease 44 inhibits cell growth by suppressing AKT signaling in non-small cell lung cancer. Kaohsiung J Med Sci. 2019;35(9):535-41. Epub 20190614. doi: 10.1002/kjm2.12096. PubMed PMID: 31197957.

75. Yang C, Zhu S, Yang H, Deng S, Fan P, Li M, et al. USP44 suppresses pancreatic cancer progression and overcomes gemcitabine resistance by deubiquitinating FBP1. Am J Cancer Res. 2019;9(8):1722-33. Epub 20190801. PubMed PMID: 31497353; PubMed Central PMCID: PMCPMC6726996.

76. Zhou J, Wang T, Qiu T, Chen Z, Ma X, Zhang L, et al. Ubiquitin-specific protease-44 inhibits the proliferation and migration of cells via inhibition of JNK pathway in clear cell renal

cell carcinoma. BMC Cancer. 2020;20(1):214. Epub 20200312. doi: 10.1186/s12885-020-6713-y. PubMed PMID: 32164618; PubMed Central PMCID: PMCPMC7068999.
77. Huang T, Zhang Q, Ren W, Yan B, Yi L, Tang T, et al. USP44 suppresses proliferation and enhances apoptosis in colorectal cancer cells by inactivating the Wnt/β-catenin pathway via Axin1 deubiquitination. Cell Biol Int. 2020;44(8):1651-9. Epub 20200421. doi: 10.1002/cbin.11358. PubMed PMID: 32285989; PubMed Central PMCID: PMCPMC7496820.
78. Chen Y, Zhao Y, Yang X, Ren X, Huang S, Gong S, et al. USP44 regulates irradiation-induced DNA double-strand break repair and suppresses tumorigenesis in nasopharyngeal carcinoma. Nat Commun. 2022;13(1):501. Epub 20220125. doi: 10.1038/s41467-022-28158-2. PubMed PMID: 35079021; PubMed Central PMCID: PMCPMC8789930.
79. Nishimura S, Oki E, Ando K, Iimori M, Nakaji Y, Nakashima Y, et al. High ubiquitin-specific protease 44 expression induces DNA aneuploidy and provides independent prognostic information in gastric cancer. Cancer Med. 2017;6(6):1453-64. Epub 20170523. doi: 10.1002/cam4.1090. PubMed PMID: 28544703; PubMed Central PMCID: PMCPMC5463085.
80. Zou Y, Qiu G, Jiang L, Cai Z, Sun W, Hu H, et al. Overexpression of ubiquitin specific proteases 44 promotes the malignancy of glioma by stabilizing tumor-promoter securin. Oncotarget. 2017;8(35):58231-46. Epub 20170322. doi: 10.18632/oncotarget.16447. PubMed PMID: 28938551; PubMed Central PMCID: PMCPMC5601647.
81. Park JM, Lee JE, Park CM, Kim JH. USP44 Promotes the Tumorigenesis of Prostate Cancer Cells through EZH2 Protein Stabilization. Mol Cells. 2019;42(1):17-27. Epub 20190102. doi: 10.14348/molcells.2018.0329. PubMed PMID: 30622230; PubMed Central PMCID: PMCPMC6354053.
82. Xiang T, Jiang HS, Zhang BT, Liu G. CircFOXO3 functions as a molecular sponge for miR-143-3p to promote the progression of gastric carcinoma via upregulating USP44. Gene. 2020;753:144798. Epub 20200520. doi: 10.1016/j.gene.2020.144798. PubMed PMID: 32445925.
83. Harripaul R, Vasli N, Mikhailov A, Rafiq MA, Mittal K, Windpassinger C, et al. Mapping autosomal recessive intellectual disability: combined microarray and exome sequencing identifies 26 novel candidate genes in 192 consanguineous families. Mol Psychiatry. 2018;23(4):973-84. Epub 20170411. doi: 10.1038/mp.2017.60. PubMed PMID: 28397838.
84. Koprulu M, Shabbir RMK, Zaman Q, Nalbant G, Malik S, Tolun A. CRADD and USP44 mutations in intellectual disability, mild lissencephaly, brain atrophy, developmental delay, strabismus, behavioural problems and skeletal anomalies. Eur J Med Genet. 2021;64(4):104181. Epub 20210227. doi: 10.1016/j.ejmg.2021.104181. PubMed PMID: 33647455.
85. Fischle W, Franz H, Jacobs SA, Allis CD, Khorasanizadeh S. Specificity of the Chromodomain Y Chromosome Family of Chromodomains for Lysine-methylated ARK(S/T) Motifs*. Journal of Biological Chemistry. 2008;283(28):19626-35. doi: https://doi.org/10.1074/jbc.M802655200.
86. Siouda M, Dujardin AD, Barbollat-Boutrand L, Mendoza-Parra MA, Gibert B, Ouzounova M, et al. CDYL2 Epigenetically Regulates MIR124 to Control NF-κB/STAT3-Dependent Breast Cancer Cell Plasticity. iScience. 2020;23(6):101141. Epub 20200506. doi: 10.1016/j.isci.2020.101141. PubMed PMID: 32450513; PubMed Central PMCID: PMCPMC7251929.
87. Yang LF, Yang F, Zhang FL, Xie YF, Hu ZX, Huang SL, et al. Discrete functional and mechanistic roles of chromodomain Y-like 2 (CDYL2) transcript variants in breast cancer growth and metastasis. Theranostics. 2020;10(12):5242-58. Epub 20200406. doi: 10.7150/thno.43744. PubMed PMID: 32373210; PubMed Central PMCID: PMCPMC7196301.

88. Chen X, Wang Z, Zhao X, Zhang L, Zhou L, Li X, et al. STAT5A modulates CDYL2/SLC7A6 pathway to inhibit the proliferation and invasion of hepatocellular carcinoma by targeting to mTORC1. Oncogene. 2022;41(17):2492-504. Epub 20220321. doi: 10.1038/s41388-022-02273-2. PubMed PMID: 35314791.

89. Buchner DA, Charrier A, Srinivasan E, Wang L, Paulsen MT, Ljungman M, et al. Zinc finger protein 407 (ZFP407) regulates insulin-stimulated glucose uptake and glucose transporter 4 (Glut4) mRNA. J Biol Chem. 2015;290(10):6376-86. Epub 20150116. doi: 10.1074/jbc.M114.623736. PubMed PMID: 25596527; PubMed Central PMCID: PMCPMC4358273.

90. Charrier A, Wang L, Stephenson EJ, Ghanta SV, Ko CW, Croniger CM, et al. Zinc finger protein 407 overexpression upregulates PPAR target gene expression and improves glucose homeostasis in mice. Am J Physiol Endocrinol Metab. 2016;311(5):E869-e80. Epub 20160913. doi: 10.1152/ajpendo.00234.2016. PubMed PMID: 27624101; PubMed Central PMCID: PMCPMC5130358.

91. Ren CM, Liang Y, Wei F, Zhang YN, Zhong SQ, Gu H, et al. Balanced translocation t(3;18)(p13;q22.3) and points mutation in the ZNF407 gene detected in patients with both moderate non-syndromic intellectual disability and autism. Biochim Biophys Acta. 2013;1832(3):431-8. Epub 20121126. doi: 10.1016/j.bbadis.2012.11.009. PubMed PMID: 23195952.

92. Kambouris M, Maroun RC, Ben-Omran T, Al-Sarraj Y, Errafii K, Ali R, et al. Mutations in zinc finger 407 [ZNF407] cause a unique autosomal recessive cognitive impairment syndrome. Orphanet J Rare Dis. 2014;9:80. Epub 20140607. doi: 10.1186/1750-1172-9-80. PubMed PMID: 24907849; PubMed Central PMCID: PMCPMC4070100.

93. Gumbiner BM. Cell adhesion: the molecular basis of tissue architecture and morphogenesis. Cell. 1996;84(3):345-57. doi: 10.1016/s0092-8674(00)81279-9. PubMed PMID: 8608588.

94. Shan WS, Tanaka H, Phillips GR, Arndt K, Yoshida M, Colman DR, et al. Functional cis-heterodimers of N- and R-cadherins. J Cell Biol. 2000;148(3):579-90. doi: 10.1083/jcb.148.3.579. PubMed PMID: 10662782; PubMed Central PMCID: PMCPMC2174798.

95. Martinez-Garay I, Gil-Sanz C, Franco SJ, Espinosa A, Molnár Z, Mueller U. Cadherin 2/4 signaling via PTP1B and catenins is crucial for nucleokinesis during radial neuronal migration in the neocortex. Development. 2016;143(12):2121-34. Epub 20160505. doi: 10.1242/dev.132456. PubMed PMID: 27151949; PubMed Central PMCID: PMCPMC4920171.

96. Matsunaga E, Nambu S, Oka M, Iriki A. Complex and dynamic expression of cadherins in the embryonic marmoset cerebral cortex. Dev Growth Differ. 2015;57(6):474-83. Epub 20150617. doi: 10.1111/dgd.12228. PubMed PMID: 26081465; PubMed Central PMCID: PMCPMC4744772.

97. Sundararajan L, Norris ML, Schöneich S, Ackley BD, Lundquist EA. The fat-like cadherin CDH-4 acts cell-non-autonomously in anterior-posterior neuroblast migration. Dev Biol. 2014;392(2):141-52. Epub 20140619. doi: 10.1016/j.ydbio.2014.06.009. PubMed PMID: 24954154; PubMed Central PMCID: PMCPMC4136450.

98. Andrews GL, Mastick GS. R-cadherin is a Pax6-regulated, growth-promoting cue for pioneer axons. J Neurosci. 2003;23(30):9873-80. doi: 10.1523/jneurosci.23-30-09873.2003. PubMed PMID: 14586016; PubMed Central PMCID: PMCPMC2080860.

99. Miotto E, Sabbioni S, Veronese A, Calin GA, Gullini S, Liboni A, et al. Frequent aberrant methylation of the CDH4 gene promoter in human colorectal and gastric cancer. Cancer Res. 2004;64(22):8156-9. doi: 10.1158/0008-5472.Can-04-3000. PubMed PMID: 15548679.

100. Du C, Huang T, Sun D, Mo Y, Feng H, Zhou X, et al. CDH4 as a novel putative tumor suppressor gene epigenetically silenced by promoter hypermethylation in nasopharyngeal

carcinoma. Cancer Lett. 2011;309(1):54-61. Epub 20110612. doi: 10.1016/j.canlet.2011.05.016. PubMed PMID: 21665361.

101. Xie J, Feng Y, Lin T, Huang XY, Gan RH, Zhao Y, et al. CDH4 suppresses the progression of salivary adenoid cystic carcinoma via E-cadherin co-expression. Oncotarget. 2016;7(50):82961-71. doi: 10.18632/oncotarget.12821. PubMed PMID: 27783992; PubMed Central PMCID: PMCPMC5347745.

102. Li Z, Su D, Ying L, Yu G, Mao W. Study on expression of CDH4 in lung cancer. World J Surg Oncol. 2017;15(1):26. Epub 20170117. doi: 10.1186/s12957-016-1083-2. PubMed PMID: 28095912; PubMed Central PMCID: PMCPMC5240236.

103. Tang Q, Lu J, Zou C, Shao Y, Chen Y, Narala S, et al. CDH4 is a novel determinant of osteosarcoma tumorigenesis and metastasis. Oncogene. 2018;37(27):3617-30. Epub 20180403. doi: 10.1038/s41388-018-0231-2. PubMed PMID: 29610525.

104. Ceresa D, Alessandrini F, Bosio L, Marubbi D, Reverberi D, Malatesta P, et al. Cdh4 Down-Regulation Impairs in Vivo Infiltration and Malignancy in Patients Derived Glioblastoma Cells. Int J Mol Sci. 2019;20(16). Epub 20190818. doi: 10.3390/ijms20164028. PubMed PMID: 31426573; PubMed Central PMCID: PMCPMC6718984.

105. Martins-Lima C, Miranda-Gonçalves V, Lobo J, Constâncio V, Leite-Silva P, Guimarães-Teixeira C, et al. Cadherin switches during epithelial-mesenchymal transition: CDH4/RCAD downregulation reduces bladder cancer progression. Cell Oncol (Dordr). 2022;45(1):135-49. Epub 20220122. doi: 10.1007/s13402-021-00657-2. PubMed PMID: 35064910.
