# Supplementary material for: Genetics of vegetarianism: A genome-wide association study
Source: PLoS One. 2023 Oct 4;18(10):e0291305. doi: 10.1371/journal.pone.0291305 (PMC10550162; doi:10.1371/journal.pone.0291305)
Supplement: S2 Table — (DOCX) [file pone.0291305.s007.docx]

| **S7 Table. Other genome-wide associations of vegetarianism-associated genes** | | |
| --- | --- | --- |
| **Gene** | **Associated Traits*** | **References*** |
| TMEM241 | Hypertriglyceridemia, body mass index (BMI), waist circumference, hip circumference, height, worry measurement, osteoarthritis. | [1-8] |
| RIOK3 | Reticulocyte count, neutrophil count, white blood cell count, waist circumference, waist-hip ratio, alcohol consumption, poultry consumption, educational attainment, lung function, television watching, serum total protein levels. | [1, 2, 4, 9-21] |
| RMC1 (C18orf8) | Body weight, adult body size, body size at age 10, BMI, waist-hip ratio, hip circumference, predicted visceral adipose tissue, HDL cholesterol, triglyceride levels, alanine aminotransferase levels, type 2 diabetes, smoking behavior, alcohol consumption, poultry consumption, television watching, educational attainment, intelligence, lung function, neutrophil count, white blood cell count, monocyte percentage of white cells, C-reactive protein levels, hand grip strength, walking pace. | [3, 4, 10-12, 15-35] |
| NPC1 | BMI, body weight, body fat percentage, body size at age 10, adult body size, predicted visceral adipose tissue, waist-hip ratio, hip circumference, metabolic syndrome, cardiovascular disease, triglyceride levels, HDL cholesterol levels, LDL cholesterol levels, apolipoprotein A1 levels, alanine aminotransferase levels, monocyte percentage of white cells, neutrophil count, granulocyte count, white blood cell count, mean sphered cell volume, mean corpuscular volume, myeloid white cell count, platelet distribution width, mental health study participation, educational attainment, household income, Alzheimer's disease, bitter alcoholic beverage consumption, chronotype, walking pace. | [1, 3, 12-14, 16, 17, 21-25, 27-30, 32, 33, 36-51] |
| VRK2 | Depression, age at first sexual intercourse, schizophrenia, waist circumference adjusted for BMI, cortical thickness, cortical surface area, educational attainment, alcohol consumption and use disorder, mathematical ability, language functional connectivity, smoking, neuroticism, epilepsy, cognitive performance, BMI, bipolar disorder, autism spectrum disorder, hip circumference adjusted for BMI, waist-hip ratio, total testosterone levels, height, well-being, lamb consumption, fish- and plant-related diet, vegetarianism†, Hirschsprung disease, mood swings, anorexia nervosa, externalizing behavior, ambidextrousness, insomnia, morningness, daytime nap, post-traumatic stress disorder. | [1-3, 7, 11, 16-18, 27, 52-94] |
| TMEM132D | Anxiety, panic disorder, serum levels of protein TMEM132D, blood protein levels, triglyceride levels, cognitive decline, sleep (1/2-day periodicity). | [95-107] |
| METAP2 | Reticulocyte count, C-reactive protein levels, gamma-glutamyl transferase levels, red cell distribution width, hemoglobin A1c levels, sex hormone-binding globulin levels, body height, blood protein levels. | [3, 12, 14, 15, 21, 34, 85, 102, 108-110] |
| USP44 | C-reactive protein levels, body height, type 2 diabetes. | [26, 51, 108, 111, 112] |
| CDYL2 | Breast cancer, alkaline phosphatase levels, male-pattern baldness, heel bone mineral density, interleukin-10 levels, systolic blood pressure, pulse pressure, monocyte count, cancer (pleiotropy), inflammatory bowel disease, physical activity. | [3, 14, 21, 110, 113-124] |
| ZNF407 | Platelet count, corneal resistance factor, facial morphology, smoking, externalizing behavior, total plasma protein levels/insomnia interaction. | [3, 10, 15, 74, 88, 125-127] |
| CDH4 | Educational attainment, mathematical ability, cognitive performance, volumetric brain MRI, household income, total PHF-tau, neurofibrillary tangles, chronic kidney disease, capecitabine-induced hand-foot syndrome in cancer, C-reactive protein levels, erosive tooth wear. | [3, 17, 18, 27, 34, 49, 128-135] |

* Most of the information in this table was obtained from the GWAS Catalog [136].

† A preliminary study (not indexed or listed in the GWAS catalog) reported a GWAS-significant association of vegetarianism with rs10189138 in the VRK2 gene [94], whereas our data show suggestive significance for this SNP with strictly defined long-term vegetarianism. That study does not provide sufficient detail on the phenotype selection, quality control, or analysis, which precludes a meaningful comparison to our findings.

**References**

1. Zhu Z, Guo Y, Shi H, Liu CL, Panganiban RA, Chung W, et al. Shared genetic and experimental links between obesity-related traits and asthma subtypes in UK Biobank. J Allergy Clin Immunol. 2020;145(2):537-49. Epub 20191024. doi: 10.1016/j.jaci.2019.09.035. PubMed PMID: 31669095; PubMed Central PMCID: PMCPMC7010560.

2. Christakoudi S, Evangelou E, Riboli E, Tsilidis KK. GWAS of allometric body-shape indices in UK Biobank identifies loci suggesting associations with morphogenesis, organogenesis, adrenal cell renewal and cancer. Sci Rep. 2021;11(1):10688. Epub 20210521. doi: 10.1038/s41598-021-89176-6. PubMed PMID: 34021172; PubMed Central PMCID: PMCPMC8139988.

3. Kichaev G, Bhatia G, Loh PR, Gazal S, Burch K, Freund MK, et al. Leveraging Polygenic Functional Enrichment to Improve GWAS Power. Am J Hum Genet. 2019;104(1):65-75. Epub 20181227. doi: 10.1016/j.ajhg.2018.11.008. PubMed PMID: 30595370; PubMed Central PMCID: PMCPMC6323418.

4. Tachmazidou I, Süveges D, Min JL, Ritchie GRS, Steinberg J, Walter K, et al. Whole-Genome Sequencing Coupled to Imputation Discovers Genetic Signals for Anthropometric Traits. Am J Hum Genet. 2017;100(6):865-84. Epub 20170525. doi: 10.1016/j.ajhg.2017.04.014. PubMed PMID: 28552196; PubMed Central PMCID: PMCPMC5473732.

5. Weissglas-Volkov D, Aguilar-Salinas CA, Nikkola E, Deere KA, Cruz-Bautista I, Arellano-Campos O, et al. Genomic study in Mexicans identifies a new locus for triglycerides and refines European lipid loci. J Med Genet. 2013;50(5):298-308. Epub 20130315. doi: 10.1136/jmedgenet-2012-101461. PubMed PMID: 23505323; PubMed Central PMCID: PMCPMC3917605.

6. Lind L. Genetic Determinants of Clustering of Cardiometabolic Risk Factors in U.K. Biobank. Metab Syndr Relat Disord. 2020;18(3):121-7. Epub 20200113. doi: 10.1089/met.2019.0096. PubMed PMID: 31928498.

7. Nagel M, Watanabe K, Stringer S, Posthuma D, van der Sluis S. Item-level analyses reveal genetic heterogeneity in neuroticism. Nat Commun. 2018;9(1):905. Epub 20180302. doi: 10.1038/s41467-018-03242-8. PubMed PMID: 29500382; PubMed Central PMCID: PMCPMC5834468.

8. Tachmazidou I, Hatzikotoulas K, Southam L, Esparza-Gordillo J, Haberland V, Zheng J, et al. Identification of new therapeutic targets for osteoarthritis through genome-wide analyses of UK Biobank data. Nat Genet. 2019;51(2):230-6. Epub 20190121. doi: 10.1038/s41588-018-0327-1. PubMed PMID: 30664745; PubMed Central PMCID: PMCPMC6400267.

9. Galvan-Femenia I, Obon-Santacana M, Pineyro D, Guindo-Martinez M, Duran X, Carreras A, et al. Multitrait genome association analysis identifies new susceptibility genes for human anthropometric variation in the GCAT cohort. J Med Genet. 2018;55(11):765-78. Epub 20180830. doi: 10.1136/jmedgenet-2018-105437. PubMed PMID: 30166351; PubMed Central PMCID: PMCPMC6252362.

10. Karlsson Linner R, Biroli P, Kong E, Meddens SFW, Wedow R, Fontana MA, et al. Genome-wide association analyses of risk tolerance and risky behaviors in over 1 million individuals identify hundreds of loci and shared genetic influences. Nat Genet. 2019;51(2):245-57. Epub 20190114. doi: 10.1038/s41588-018-0309-3. PubMed PMID: 30643258; PubMed Central PMCID: PMCPMC6713272.

11. Niarchou M, Byrne EM, Trzaskowski M, Sidorenko J, Kemper KE, McGrath JJ, et al. Genome-wide association study of dietary intake in the UK biobank study and its associations with schizophrenia and other traits. Transl Psychiatry. 2020;10(1):51. Epub 20200203. doi: 10.1038/s41398-020-0688-y. PubMed PMID: 32066663; PubMed Central PMCID: PMCPMC7026164.

12. Vuckovic D, Bao EL, Akbari P, Lareau CA, Mousas A, Jiang T, et al. The Polygenic and Monogenic Basis of Blood Traits and Diseases. Cell. 2020;182(5):1214-31.e11. doi: 10.1016/j.cell.2020.08.008. PubMed PMID: 32888494; PubMed Central PMCID: PMCPMC7482360.

13. Barton AR, Sherman MA, Mukamel RE, Loh PR. Whole-exome imputation within UK Biobank powers rare coding variant association and fine-mapping analyses. Nat Genet. 2021;53(8):1260-9. Epub 20210705. doi: 10.1038/s41588-021-00892-1. PubMed PMID: 34226706; PubMed Central PMCID: PMCPMC8349845.

14. Astle WJ, Elding H, Jiang T, Allen D, Ruklisa D, Mann AL, et al. The Allelic Landscape of Human Blood Cell Trait Variation and Links to Common Complex Disease. Cell. 2016;167(5):1415-29.e19. doi: 10.1016/j.cell.2016.10.042. PubMed PMID: 27863252; PubMed Central PMCID: PMCPMC5300907.

15. Chen MH, Raffield LM, Mousas A, Sakaue S, Huffman JE, Moscati A, et al. Trans-ethnic and Ancestry-Specific Blood-Cell Genetics in 746,667 Individuals from 5 Global Populations. Cell. 2020;182(5):1198-213.e14. doi: 10.1016/j.cell.2020.06.045. PubMed PMID: 32888493; PubMed Central PMCID: PMCPMC7480402.

16. Pulit SL, Stoneman C, Morris AP, Wood AR, Glastonbury CA, Tyrrell J, et al. Meta-analysis of genome-wide association studies for body fat distribution in 694 649 individuals of European ancestry. Hum Mol Genet. 2019;28(1):166-74. doi: 10.1093/hmg/ddy327. PubMed PMID: 30239722; PubMed Central PMCID: PMCPMC6298238.

17. Lee JJ, Wedow R, Okbay A, Kong E, Maghzian O, Zacher M, et al. Gene discovery and polygenic prediction from a genome-wide association study of educational attainment in 1.1 million individuals. Nat Genet. 2018;50(8):1112-21. Epub 20180723. doi: 10.1038/s41588-018-0147-3. PubMed PMID: 30038396; PubMed Central PMCID: PMCPMC6393768.

18. Okbay A, Wu Y, Wang N, Jayashankar H, Bennett M, Nehzati SM, et al. Polygenic prediction of educational attainment within and between families from genome-wide association analyses in 3 million individuals. Nat Genet. 2022;54(4):437-49. Epub 20220331. doi: 10.1038/s41588-022-01016-z. PubMed PMID: 35361970; PubMed Central PMCID: PMCPMC9005349.

19. Shrine N, Guyatt AL, Erzurumluoglu AM, Jackson VE, Hobbs BD, Melbourne CA, et al. New genetic signals for lung function highlight pathways and chronic obstructive pulmonary disease associations across multiple ancestries. Nat Genet. 2019;51(3):481-93. Epub 20190225. doi: 10.1038/s41588-018-0321-7. PubMed PMID: 30804560; PubMed Central PMCID: PMCPMC6397078.

20. van de Vegte YJ, Said MA, Rienstra M, van der Harst P, Verweij N. Genome-wide association studies and Mendelian randomization analyses for leisure sedentary behaviours. Nat Commun. 2020;11(1):1770. Epub 20200421. doi: 10.1038/s41467-020-15553-w. PubMed PMID: 32317632; PubMed Central PMCID: PMCPMC7174427.

21. Sakaue S, Kanai M, Tanigawa Y, Karjalainen J, Kurki M, Koshiba S, et al. A cross-population atlas of genetic associations for 220 human phenotypes. Nat Genet. 2021;53(10):1415-24. Epub 20210930. doi: 10.1038/s41588-021-00931-x. PubMed PMID: 34594039.

22. Justice AE, Winkler TW, Feitosa MF, Graff M, Fisher VA, Young K, et al. Genome-wide meta-analysis of 241,258 adults accounting for smoking behaviour identifies novel loci for obesity traits. Nat Commun. 2017;8:14977. Epub 20170426. doi: 10.1038/ncomms14977. PubMed PMID: 28443625; PubMed Central PMCID: PMCPMC5414044.

23. Shungin D, Winkler TW, Croteau-Chonka DC, Ferreira T, Locke AE, Mägi R, et al. New genetic loci link adipose and insulin biology to body fat distribution. Nature. 2015;518(7538):187-96. doi: 10.1038/nature14132. PubMed PMID: 25673412; PubMed Central PMCID: PMCPMC4338562.

24. Locke AE, Kahali B, Berndt SI, Justice AE, Pers TH, Day FR, et al. Genetic studies of body mass index yield new insights for obesity biology. Nature. 2015;518(7538):197-206. doi: 10.1038/nature14177. PubMed PMID: 25673413; PubMed Central PMCID: PMCPMC4382211.

25. Karlsson T, Rask-Andersen M, Pan G, Höglund J, Wadelius C, Ek WE, et al. Contribution of genetics to visceral adiposity and its relation to cardiovascular and metabolic disease. Nat Med. 2019;25(9):1390-5. Epub 20190909. doi: 10.1038/s41591-019-0563-7. PubMed PMID: 31501611.

26. Vujkovic M, Keaton JM, Lynch JA, Miller DR, Zhou J, Tcheandjieu C, et al. Discovery of 318 new risk loci for type 2 diabetes and related vascular outcomes among 1.4 million participants in a multi-ancestry meta-analysis. Nat Genet. 2020;52(7):680-91. Epub 20200615. doi: 10.1038/s41588-020-0637-y. PubMed PMID: 32541925; PubMed Central PMCID: PMCPMC7343592.

27. Pasman JA, Demange PA, Guloksuz S, Willemsen AHM, Abdellaoui A, Ten Have M, et al. Genetic Risk for Smoking: Disentangling Interplay Between Genes and Socioeconomic Status. Behav Genet. 2022;52(2):92-107. Epub 20211202. doi: 10.1007/s10519-021-10094-4. PubMed PMID: 34855049; PubMed Central PMCID: PMCPMC8860781.

28. Akiyama M, Okada Y, Kanai M, Takahashi A, Momozawa Y, Ikeda M, et al. Genome-wide association study identifies 112 new loci for body mass index in the Japanese population. Nat Genet. 2017;49(10):1458-67. Epub 20170911. doi: 10.1038/ng.3951. PubMed PMID: 28892062.

29. Hoffmann TJ, Choquet H, Yin J, Banda Y, Kvale MN, Glymour M, et al. A Large Multiethnic Genome-Wide Association Study of Adult Body Mass Index Identifies Novel Loci. Genetics. 2018;210(2):499-515. Epub 20180814. doi: 10.1534/genetics.118.301479. PubMed PMID: 30108127; PubMed Central PMCID: PMCPMC6216593.

30. Zhong VW, Kuang A, Danning RD, Kraft P, van Dam RM, Chasman DI, et al. A genome-wide association study of bitter and sweet beverage consumption. Hum Mol Genet. 2019;28(14):2449-57. doi: 10.1093/hmg/ddz061. PubMed PMID: 31046077; PubMed Central PMCID: PMCPMC6606847.

31. Tikkanen E, Gustafsson S, Amar D, Shcherbina A, Waggott D, Ashley EA, et al. Biological Insights Into Muscular Strength: Genetic Findings in the UK Biobank. Sci Rep. 2018;8(1):6451. Epub 20180424. doi: 10.1038/s41598-018-24735-y. PubMed PMID: 29691431; PubMed Central PMCID: PMCPMC5915424.

32. Richardson TG, Sanderson E, Elsworth B, Tilling K, Davey Smith G. Use of genetic variation to separate the effects of early and later life adiposity on disease risk: mendelian randomisation study. Bmj. 2020;369:m1203. Epub 20200506. doi: 10.1136/bmj.m1203. PubMed PMID: 32376654; PubMed Central PMCID: PMCPMC7201936.

33. Timmins IR, Zaccardi F, Nelson CP, Franks PW, Yates T, Dudbridge F. Genome-wide association study of self-reported walking pace suggests beneficial effects of brisk walking on health and survival. Commun Biol. 2020;3(1):634. Epub 20201030. doi: 10.1038/s42003-020-01357-7. PubMed PMID: 33128006; PubMed Central PMCID: PMCPMC7599247.

34. Han X, Ong JS, An J, Hewitt AW, Gharahkhani P, MacGregor S. Using Mendelian randomization to evaluate the causal relationship between serum C-reactive protein levels and age-related macular degeneration. Eur J Epidemiol. 2020;35(2):139-46. Epub 20200103. doi: 10.1007/s10654-019-00598-z. PubMed PMID: 31900758.

35. Hill WD, Marioni RE, Maghzian O, Ritchie SJ, Hagenaars SP, McIntosh AM, et al. A combined analysis of genetically correlated traits identifies 187 loci and a role for neurogenesis and myelination in intelligence. Mol Psychiatry. 2019;24(2):169-81. Epub 20180111. doi: 10.1038/s41380-017-0001-5. PubMed PMID: 29326435; PubMed Central PMCID: PMCPMC6344370.

36. Winkler TW, Justice AE, Graff M, Barata L, Feitosa MF, Chu S, et al. The Influence of Age and Sex on Genetic Associations with Adult Body Size and Shape: A Large-Scale Genome-Wide Interaction Study. PLoS Genet. 2015;11(10):e1005378. Epub 20151001. doi: 10.1371/journal.pgen.1005378. PubMed PMID: 26426971; PubMed Central PMCID: PMCPMC4591371.

37. Wood AR, Tyrrell J, Beaumont R, Jones SE, Tuke MA, Ruth KS, et al. Variants in the FTO and CDKAL1 loci have recessive effects on risk of obesity and type 2 diabetes, respectively. Diabetologia. 2016;59(6):1214-21. Epub 20160310. doi: 10.1007/s00125-016-3908-5. PubMed PMID: 26961502; PubMed Central PMCID: PMCPMC4869698.

38. Turcot V, Lu Y, Highland HM, Schurmann C, Justice AE, Fine RS, et al. Protein-altering variants associated with body mass index implicate pathways that control energy intake and expenditure in obesity. Nat Genet. 2018;50(1):26-41. Epub 20171222. doi: 10.1038/s41588-017-0011-x. PubMed PMID: 29273807; PubMed Central PMCID: PMCPMC5945951.

39. Zhuang Z, Yao M, Wong JYY, Liu Z, Huang T. Shared genetic etiology and causality between body fat percentage and cardiovascular diseases: a large-scale genome-wide cross-trait analysis. BMC Med. 2021;19(1):100. Epub 20210429. doi: 10.1186/s12916-021-01972-z. PubMed PMID: 33910581; PubMed Central PMCID: PMCPMC8082910.

40. Martin S, Cule M, Basty N, Tyrrell J, Beaumont RN, Wood AR, et al. Genetic Evidence for Different Adiposity Phenotypes and Their Opposing Influences on Ectopic Fat and Risk of Cardiometabolic Disease. Diabetes. 2021;70(8):1843-56. Epub 20210512. doi: 10.2337/db21-0129. PubMed PMID: 33980691.

41. Richardson TG, Sanderson E, Palmer TM, Ala-Korpela M, Ference BA, Davey Smith G, et al. Evaluating the relationship between circulating lipoprotein lipids and apolipoproteins with risk of coronary heart disease: A multivariable Mendelian randomisation analysis. PLoS Med. 2020;17(3):e1003062. Epub 20200323. doi: 10.1371/journal.pmed.1003062. PubMed PMID: 32203549; PubMed Central PMCID: PMCPMC7089422.

42. Ripatti P, Rämö JT, Mars NJ, Fu Y, Lin J, Söderlund S, et al. Polygenic Hyperlipidemias and Coronary Artery Disease Risk. Circ Genom Precis Med. 2020;13(2):e002725. Epub 20200310. doi: 10.1161/circgen.119.002725. PubMed PMID: 32154731; PubMed Central PMCID: PMCPMC7176338.

43. Klarin D, Damrauer SM, Cho K, Sun YV, Teslovich TM, Honerlaw J, et al. Genetics of blood lipids among ~300,000 multi-ethnic participants of the Million Veteran Program. Nat Genet. 2018;50(11):1514-23. Epub 20181001. doi: 10.1038/s41588-018-0222-9. PubMed PMID: 30275531; PubMed Central PMCID: PMCPMC6521726.

44. Kulminski AM, Loiko E, Loika Y, Culminskaya I. Pleiotropic predisposition to Alzheimer's disease and educational attainment: insights from the summary statistics analysis. Geroscience. 2022;44(1):265-80. Epub 20211106. doi: 10.1007/s11357-021-00484-1. PubMed PMID: 34743297; PubMed Central PMCID: PMCPMC8572080.

45. Lam M, Hill WD, Trampush JW, Yu J, Knowles E, Davies G, et al. Pleiotropic Meta-Analysis of Cognition, Education, and Schizophrenia Differentiates Roles of Early Neurodevelopmental and Adult Synaptic Pathways. Am J Hum Genet. 2019;105(2):334-50. doi: 10.1016/j.ajhg.2019.06.012. PubMed PMID: 31374203; PubMed Central PMCID: PMCPMC6699140.

46. Lind L. Genome-Wide Association Study of the Metabolic Syndrome in UK Biobank. Metab Syndr Relat Disord. 2019;17(10):505-11. Epub 20191007. doi: 10.1089/met.2019.0070. PubMed PMID: 31589552.

47. Adams MJ, Hill WD, Howard DM, Dashti HS, Davis KAS, Campbell A, et al. Factors associated with sharing e-mail information and mental health survey participation in large population cohorts. Int J Epidemiol. 2020;49(2):410-21. doi: 10.1093/ije/dyz134. PubMed PMID: 31263887; PubMed Central PMCID: PMCPMC7266553.

48. Jones SE, Lane JM, Wood AR, van Hees VT, Tyrrell J, Beaumont RN, et al. Genome-wide association analyses of chronotype in 697,828 individuals provides insights into circadian rhythms. Nat Commun. 2019;10(1):343. Epub 20190129. doi: 10.1038/s41467-018-08259-7. PubMed PMID: 30696823; PubMed Central PMCID: PMCPMC6351539.

49. Hill WD, Davies NM, Ritchie SJ, Skene NG, Bryois J, Bell S, et al. Genome-wide analysis identifies molecular systems and 149 genetic loci associated with income. Nat Commun. 2019;10(1):5741. Epub 20191216. doi: 10.1038/s41467-019-13585-5. PubMed PMID: 31844048; PubMed Central PMCID: PMCPMC6915786.

50. Klimentidis YC, Arora A, Newell M, Zhou J, Ordovas JM, Renquist BJ, et al. Phenotypic and Genetic Characterization of Lower LDL Cholesterol and Increased Type 2 Diabetes Risk in the UK Biobank. Diabetes. 2020;69(10):2194-205. Epub 20200603. doi: 10.2337/db19-1134. PubMed PMID: 32493714; PubMed Central PMCID: PMCPMC7506834.

51. Sinnott-Armstrong N, Tanigawa Y, Amar D, Mars N, Benner C, Aguirre M, et al. Genetics of 35 blood and urine biomarkers in the UK Biobank. Nat Genet. 2021;53(2):185-94. Epub 20210118. doi: 10.1038/s41588-020-00757-z. PubMed PMID: 33462484; PubMed Central PMCID: PMCPMC7867639.

52. Li X, Luo Z, Gu C, Hall LS, McIntosh AM, Zeng Y, et al. Common variants on 6q16.2, 12q24.31 and 16p13.3 are associated with major depressive disorder. Neuropsychopharmacology. 2018;43(10):2146-53. Epub 20180427. doi: 10.1038/s41386-018-0078-9. PubMed PMID: 29728651; PubMed Central PMCID: PMCPMC6098070.

53. Nagel M, Jansen PR, Stringer S, Watanabe K, de Leeuw CA, Bryois J, et al. Meta-analysis of genome-wide association studies for neuroticism in 449,484 individuals identifies novel genetic loci and pathways. Nat Genet. 2018;50(7):920-7. Epub 20180625. doi: 10.1038/s41588-018-0151-7. PubMed PMID: 29942085.

54. Howard DM, Adams MJ, Clarke TK, Hafferty JD, Gibson J, Shirali M, et al. Genome-wide meta-analysis of depression identifies 102 independent variants and highlights the importance of the prefrontal brain regions. Nat Neurosci. 2019;22(3):343-52. Epub 20190204. doi: 10.1038/s41593-018-0326-7. PubMed PMID: 30718901; PubMed Central PMCID: PMCPMC6522363.

55. Hyde CL, Nagle MW, Tian C, Chen X, Paciga SA, Wendland JR, et al. Identification of 15 genetic loci associated with risk of major depression in individuals of European descent. Nat Genet. 2016;48(9):1031-6. Epub 20160801. doi: 10.1038/ng.3623. PubMed PMID: 27479909; PubMed Central PMCID: PMCPMC5706769.

56. Thorp JG, Campos AI, Grotzinger AD, Gerring ZF, An J, Ong JS, et al. Symptom-level modelling unravels the shared genetic architecture of anxiety and depression. Nat Hum Behav. 2021;5(10):1432-42. Epub 20210415. doi: 10.1038/s41562-021-01094-9. PubMed PMID: 33859377.

57. Cai N, Revez JA, Adams MJ, Andlauer TFM, Breen G, Byrne EM, et al. Minimal phenotyping yields genome-wide association signals of low specificity for major depression. Nat Genet. 2020;52(4):437-47. Epub 20200330. doi: 10.1038/s41588-020-0594-5. PubMed PMID: 32231276; PubMed Central PMCID: PMCPMC7906795.

58. Genomic Relationships, Novel Loci, and Pleiotropic Mechanisms across Eight Psychiatric Disorders. Cell. 2019;179(7):1469-82.e11. doi: 10.1016/j.cell.2019.11.020. PubMed PMID: 31835028; PubMed Central PMCID: PMCPMC7077032.

59. Mills MC, Tropf FC, Brazel DM, van Zuydam N, Vaez A, Pers TH, et al. Identification of 371 genetic variants for age at first sex and birth linked to externalising behaviour. Nat Hum Behav. 2021;5(12):1717-30. Epub 20210701. doi: 10.1038/s41562-021-01135-3. PubMed PMID: 34211149; PubMed Central PMCID: PMCPMC7612120.

60. Stefansson H, Ophoff RA, Steinberg S, Andreassen OA, Cichon S, Rujescu D, et al. Common variants conferring risk of schizophrenia. Nature. 2009;460(7256):744-7. Epub 20090701. doi: 10.1038/nature08186. PubMed PMID: 19571808; PubMed Central PMCID: PMCPMC3077530.

61. Lam M, Chen CY, Li Z, Martin AR, Bryois J, Ma X, et al. Comparative genetic architectures of schizophrenia in East Asian and European populations. Nat Genet. 2019;51(12):1670-8. Epub 20191118. doi: 10.1038/s41588-019-0512-x. PubMed PMID: 31740837; PubMed Central PMCID: PMCPMC6885121.

62. Yao X, Glessner JT, Li J, Qi X, Hou X, Zhu C, et al. Integrative analysis of genome-wide association studies identifies novel loci associated with neuropsychiatric disorders. Transl Psychiatry. 2021;11(1):69. Epub 20210121. doi: 10.1038/s41398-020-01195-5. PubMed PMID: 33479212; PubMed Central PMCID: PMCPMC7820351.

63. Yu H, Yan H, Li J, Li Z, Zhang X, Ma Y, et al. Common variants on 2p16.1, 6p22.1 and 10q24.32 are associated with schizophrenia in Han Chinese population. Mol Psychiatry. 2017;22(7):954-60. Epub 20161206. doi: 10.1038/mp.2016.212. PubMed PMID: 27922604.

64. Pardiñas AF, Holmans P, Pocklington AJ, Escott-Price V, Ripke S, Carrera N, et al. Common schizophrenia alleles are enriched in mutation-intolerant genes and in regions under strong background selection. Nat Genet. 2018;50(3):381-9. Epub 20180226. doi: 10.1038/s41588-018-0059-2. PubMed PMID: 29483656; PubMed Central PMCID: PMCPMC5918692.

65. Li Z, Chen J, Yu H, He L, Xu Y, Zhang D, et al. Genome-wide association analysis identifies 30 new susceptibility loci for schizophrenia. Nat Genet. 2017;49(11):1576-83. Epub 20171009. doi: 10.1038/ng.3973. PubMed PMID: 28991256.

66. Meta-analysis of GWAS of over 16,000 individuals with autism spectrum disorder highlights a novel locus at 10q24.32 and a significant overlap with schizophrenia. Mol Autism. 2017;8:21. Epub 20170522. doi: 10.1186/s13229-017-0137-9. PubMed PMID: 28540026; PubMed Central PMCID: PMCPMC5441062.

67. Ikeda M, Takahashi A, Kamatani Y, Momozawa Y, Saito T, Kondo K, et al. Genome-Wide Association Study Detected Novel Susceptibility Genes for Schizophrenia and Shared Trans-Populations/Diseases Genetic Effect. Schizophr Bull. 2019;45(4):824-34. doi: 10.1093/schbul/sby140. PubMed PMID: 30285260; PubMed Central PMCID: PMCPMC6581133.

68. Wu Y, Cao H, Baranova A, Huang H, Li S, Cai L, et al. Multi-trait analysis for genome-wide association study of five psychiatric disorders. Transl Psychiatry. 2020;10(1):209. Epub 20200630. doi: 10.1038/s41398-020-00902-6. PubMed PMID: 32606422; PubMed Central PMCID: PMCPMC7326916.

69. Goes FS, McGrath J, Avramopoulos D, Wolyniec P, Pirooznia M, Ruczinski I, et al. Genome-wide association study of schizophrenia in Ashkenazi Jews. Am J Med Genet B Neuropsychiatr Genet. 2015;168(8):649-59. Epub 20150721. doi: 10.1002/ajmg.b.32349. PubMed PMID: 26198764.

70. Shadrin AA, Kaufmann T, van der Meer D, Palmer CE, Makowski C, Loughnan R, et al. Vertex-wise multivariate genome-wide association study identifies 780 unique genetic loci associated with cortical morphology. Neuroimage. 2021;244:118603. Epub 20210921. doi: 10.1016/j.neuroimage.2021.118603. PubMed PMID: 34560273; PubMed Central PMCID: PMCPMC8785963.

71. Wendt FR, Pathak GA, Lencz T, Krystal JH, Gelernter J, Polimanti R. Multivariate genome-wide analysis of education, socioeconomic status and brain phenome. Nat Hum Behav. 2021;5(4):482-96. Epub 20201221. doi: 10.1038/s41562-020-00980-y. PubMed PMID: 33349686; PubMed Central PMCID: PMCPMC8068566.

72. Zhou H, Sealock JM, Sanchez-Roige S, Clarke TK, Levey DF, Cheng Z, et al. Genome-wide meta-analysis of problematic alcohol use in 435,563 individuals yields insights into biology and relationships with other traits. Nat Neurosci. 2020;23(7):809-18. Epub 20200525. doi: 10.1038/s41593-020-0643-5. PubMed PMID: 32451486; PubMed Central PMCID: PMCPMC7485556.

73. Kranzler HR, Zhou H, Kember RL, Vickers Smith R, Justice AC, Damrauer S, et al. Genome-wide association study of alcohol consumption and use disorder in 274,424 individuals from multiple populations. Nat Commun. 2019;10(1):1499. Epub 20190402. doi: 10.1038/s41467-019-09480-8. PubMed PMID: 30940813; PubMed Central PMCID: PMCPMC6445072.

74. Liu M, Jiang Y, Wedow R, Li Y, Brazel DM, Chen F, et al. Association studies of up to 1.2 million individuals yield new insights into the genetic etiology of tobacco and alcohol use. Nat Genet. 2019;51(2):237-44. Epub 20190114. doi: 10.1038/s41588-018-0307-5. PubMed PMID: 30643251; PubMed Central PMCID: PMCPMC6358542.

75. Mekki Y, Guillemot V, Lemaître H, Carrión-Castillo A, Forkel S, Frouin V, et al. The genetic architecture of language functional connectivity. Neuroimage. 2022;249:118795. Epub 20211218. doi: 10.1016/j.neuroimage.2021.118795. PubMed PMID: 34929384.

76. Xu K, Li B, McGinnis KA, Vickers-Smith R, Dao C, Sun N, et al. Genome-wide association study of smoking trajectory and meta-analysis of smoking status in 842,000 individuals. Nat Commun. 2020;11(1):5302. Epub 20201020. doi: 10.1038/s41467-020-18489-3. PubMed PMID: 33082346; PubMed Central PMCID: PMCPMC7598939.

77. Turley P, Walters RK, Maghzian O, Okbay A, Lee JJ, Fontana MA, et al. Multi-trait analysis of genome-wide association summary statistics using MTAG. Nat Genet. 2018;50(2):229-37. Epub 20180101. doi: 10.1038/s41588-017-0009-4. PubMed PMID: 29292387; PubMed Central PMCID: PMCPMC5805593.

78. Smith DJ, Escott-Price V, Davies G, Bailey ME, Colodro-Conde L, Ward J, et al. Genome-wide analysis of over 106 000 individuals identifies 9 neuroticism-associated loci. Mol Psychiatry. 2016;21(6):749-57. Epub 20160412. doi: 10.1038/mp.2016.49. PubMed PMID: 27067015; PubMed Central PMCID: PMCPMC4879189.

79. Baselmans BML, Jansen R, Ip HF, van Dongen J, Abdellaoui A, van de Weijer MP, et al. Multivariate genome-wide analyses of the well-being spectrum. Nat Genet. 2019;51(3):445-51. Epub 20190114. doi: 10.1038/s41588-018-0320-8. PubMed PMID: 30643256.

80. Luciano M, Hagenaars SP, Davies G, Hill WD, Clarke TK, Shirali M, et al. Association analysis in over 329,000 individuals identifies 116 independent variants influencing neuroticism. Nat Genet. 2018;50(1):6-11. Epub 20171218. doi: 10.1038/s41588-017-0013-8. PubMed PMID: 29255261; PubMed Central PMCID: PMCPMC5985926.

81. Zhang F, Cao H, Baranova A. Shared Genetic Liability and Causal Associations Between Major Depressive Disorder and Cardiovascular Diseases. Front Cardiovasc Med. 2021;8:735136. Epub 20211111. doi: 10.3389/fcvm.2021.735136. PubMed PMID: 34859065; PubMed Central PMCID: PMCPMC8631916.

82. Genome-wide mega-analysis identifies 16 loci and highlights diverse biological mechanisms in the common epilepsies. Nat Commun. 2018;9(1):5269. Epub 20181210. doi: 10.1038/s41467-018-07524-z. PubMed PMID: 30531953; PubMed Central PMCID: PMCPMC6288131.

83. Steffens M, Leu C, Ruppert AK, Zara F, Striano P, Robbiano A, et al. Genome-wide association analysis of genetic generalized epilepsies implicates susceptibility loci at 1q43, 2p16.1, 2q22.3 and 17q21.32. Hum Mol Genet. 2012;21(24):5359-72. Epub 20120904. doi: 10.1093/hmg/dds373. PubMed PMID: 22949513.

84. Genetic determinants of common epilepsies: a meta-analysis of genome-wide association studies. Lancet Neurol. 2014;13(9):893-903. Epub 20140730. doi: 10.1016/s1474-4422(14)70171-1. PubMed PMID: 25087078; PubMed Central PMCID: PMCPMC4189926.

85. Ruth KS, Day FR, Tyrrell J, Thompson DJ, Wood AR, Mahajan A, et al. Using human genetics to understand the disease impacts of testosterone in men and women. Nat Med. 2020;26(2):252-8. Epub 20200210. doi: 10.1038/s41591-020-0751-5. PubMed PMID: 32042192; PubMed Central PMCID: PMCPMC7025895.

86. Fang H, Hui Q, Lynch J, Honerlaw J, Assimes TL, Huang J, et al. Harmonizing Genetic Ancestry and Self-identified Race/Ethnicity in Genome-wide Association Studies. Am J Hum Genet. 2019;105(4):763-72. Epub 20190926. doi: 10.1016/j.ajhg.2019.08.012. PubMed PMID: 31564439; PubMed Central PMCID: PMCPMC6817526.

87. Tang CS, Gui H, Kapoor A, Kim JH, Luzón-Toro B, Pelet A, et al. Trans-ethnic meta-analysis of genome-wide association studies for Hirschsprung disease. Hum Mol Genet. 2016;25(23):5265-75. doi: 10.1093/hmg/ddw333. PubMed PMID: 27702942; PubMed Central PMCID: PMCPMC6078638.

88. Karlsson Linnér R, Mallard TT, Barr PB, Sanchez-Roige S, Madole JW, Driver MN, et al. Multivariate analysis of 1.5 million people identifies genetic associations with traits related to self-regulation and addiction. Nat Neurosci. 2021;24(10):1367-76. Epub 20210826. doi: 10.1038/s41593-021-00908-3. PubMed PMID: 34446935; PubMed Central PMCID: PMCPMC8484054.

89. Cuellar-Partida G, Tung JY, Eriksson N, Albrecht E, Aliev F, Andreassen OA, et al. Genome-wide association study identifies 48 common genetic variants associated with handedness. Nat Hum Behav. 2021;5(1):59-70. Epub 20200928. doi: 10.1038/s41562-020-00956-y. PubMed PMID: 32989287; PubMed Central PMCID: PMCPMC7116623.

90. Lane JM, Jones SE, Dashti HS, Wood AR, Aragam KG, van Hees VT, et al. Biological and clinical insights from genetics of insomnia symptoms. Nat Genet. 2019;51(3):387-93. Epub 20190225. doi: 10.1038/s41588-019-0361-7. PubMed PMID: 30804566; PubMed Central PMCID: PMCPMC6415688.

91. Jansen PR, Watanabe K, Stringer S, Skene N, Bryois J, Hammerschlag AR, et al. Genome-wide analysis of insomnia in 1,331,010 individuals identifies new risk loci and functional pathways. Nat Genet. 2019;51(3):394-403. Epub 20190225. doi: 10.1038/s41588-018-0333-3. PubMed PMID: 30804565.

92. Dashti HS, Daghlas I, Lane JM, Huang Y, Udler MS, Wang H, et al. Genetic determinants of daytime napping and effects on cardiometabolic health. Nat Commun. 2021;12(1):900. Epub 20210210. doi: 10.1038/s41467-020-20585-3. PubMed PMID: 33568662; PubMed Central PMCID: PMCPMC7876146.

93. Wendt FR, Pathak GA, Deak JD, De Angelis F, Koller D, Cabrera-Mendoza B, et al. Using phenotype risk scores to enhance gene discovery for generalized anxiety disorder and posttraumatic stress disorder. Mol Psychiatry. 2022;27(4):2206-15. Epub 20220218. doi: 10.1038/s41380-022-01469-y. PubMed PMID: 35181757; PubMed Central PMCID: PMCPMC9133008.

94. Fensom G, Smith-Byrne K, Andrews C, Key T, Travis R. Genome-wide association study of vegetarianism in UK Biobank identifies association with VRK2 [version 1; peer review: 3 approved with reservations]. Wellcome Open Research. 2020;5(291). doi: 10.12688/wellcomeopenres.16396.1.

95. Erhardt A, Czibere L, Roeske D, Lucae S, Unschuld PG, Ripke S, et al. TMEM132D, a new candidate for anxiety phenotypes: evidence from human and mouse studies. Mol Psychiatry. 2011;16(6):647-63. Epub 20100406. doi: 10.1038/mp.2010.41. PubMed PMID: 20368705.

96. Quast C, Altmann A, Weber P, Arloth J, Bader D, Heck A, et al. Rare variants in TMEM132D in a case-control sample for panic disorder. Am J Med Genet B Neuropsychiatr Genet. 2012;159b(8):896-907. Epub 20120822. doi: 10.1002/ajmg.b.32096. PubMed PMID: 22911938.

97. Erhardt A, Akula N, Schumacher J, Czamara D, Karbalai N, Müller-Myhsok B, et al. Replication and meta-analysis of TMEM132D gene variants in panic disorder. Transl Psychiatry. 2012;2(9):e156. Epub 20120904. doi: 10.1038/tp.2012.85. PubMed PMID: 22948381; PubMed Central PMCID: PMCPMC3565207.

98. Howe AS, Buttenschøn HN, Bani-Fatemi A, Maron E, Otowa T, Erhardt A, et al. Candidate genes in panic disorder: meta-analyses of 23 common variants in major anxiogenic pathways. Mol Psychiatry. 2016;21(5):665-79. Epub 20150922. doi: 10.1038/mp.2015.138. PubMed PMID: 26390831.

99. Shimada-Sugimoto M, Otowa T, Miyagawa T, Khor SS, Omae Y, Toyo-Oka L, et al. Polymorphisms in the TMEM132D region are associated with panic disorder in HLA-DRB1*13:02-negative individuals of a Japanese population. Hum Genome Var. 2016;3:16001. Epub 20160225. doi: 10.1038/hgv.2016.1. PubMed PMID: 27081567; PubMed Central PMCID: PMCPMC4766370.

100. Hodgson K, Almasy L, Knowles EE, Kent JW, Curran JE, Dyer TD, et al. Genome-wide significant loci for addiction and anxiety. Eur Psychiatry. 2016;36:47-54. Epub 20160616. doi: 10.1016/j.eurpsy.2016.03.004. PubMed PMID: 27318301; PubMed Central PMCID: PMCPMC5483998.

101. Gudjonsson A, Gudmundsdottir V, Axelsson GT, Gudmundsson EF, Jonsson BG, Launer LJ, et al. A genome-wide association study of serum proteins reveals shared loci with common diseases. Nat Commun. 2022;13(1):480. Epub 20220125. doi: 10.1038/s41467-021-27850-z. PubMed PMID: 35078996; PubMed Central PMCID: PMCPMC8789779.

102. Emilsson V, Ilkov M, Lamb JR, Finkel N, Gudmundsson EF, Pitts R, et al. Co-regulatory networks of human serum proteins link genetics to disease. Science. 2018;361(6404):769-73. Epub 20180802. doi: 10.1126/science.aaq1327. PubMed PMID: 30072576; PubMed Central PMCID: PMCPMC6190714.

103. Sun BB, Maranville JC, Peters JE, Stacey D, Staley JR, Blackshaw J, et al. Genomic atlas of the human plasma proteome. Nature. 2018;558(7708):73-9. Epub 20180606. doi: 10.1038/s41586-018-0175-2. PubMed PMID: 29875488; PubMed Central PMCID: PMCPMC6697541.

104. Wan JY, Goodman DL, Willems EL, Freedland AR, Norden-Krichmar TM, Santorico SA, et al. Genome-wide association analysis of metabolic syndrome quantitative traits in the GENNID multiethnic family study. Diabetol Metab Syndr. 2021;13(1):59. Epub 20210601. doi: 10.1186/s13098-021-00670-3. PubMed PMID: 34074324; PubMed Central PMCID: PMCPMC8170963.

105. Li QS, Parrado AR, Samtani MN, Narayan VA. Variations in the FRA10AC1 Fragile Site and 15q21 Are Associated with Cerebrospinal Fluid Aβ1-42 Level. PLoS One. 2015;10(8):e0134000. Epub 20150807. doi: 10.1371/journal.pone.0134000. PubMed PMID: 26252872; PubMed Central PMCID: PMCPMC4529186.

106. Sherva R, Gross A, Mukherjee S, Koesterer R, Amouyel P, Bellenguez C, et al. Genome-wide association study of rate of cognitive decline in Alzheimer's disease patients identifies novel genes and pathways. Alzheimers Dement. 2020;16(8):1134-45. Epub 20200623. doi: 10.1002/alz.12106. PubMed PMID: 32573913; PubMed Central PMCID: PMCPMC7924136.

107. Li X, Zhao H. Automated feature extraction from population wearable device data identified novel loci associated with sleep and circadian rhythms. PLoS Genet. 2020;16(10):e1009089. Epub 20201019. doi: 10.1371/journal.pgen.1009089. PubMed PMID: 33075057; PubMed Central PMCID: PMCPMC7595622.

108. Ligthart S, Vaez A, Võsa U, Stathopoulou MG, de Vries PS, Prins BP, et al. Genome Analyses of >200,000 Individuals Identify 58 Loci for Chronic Inflammation and Highlight Pathways that Link Inflammation and Complex Disorders. Am J Hum Genet. 2018;103(5):691-706. doi: 10.1016/j.ajhg.2018.09.009. PubMed PMID: 30388399; PubMed Central PMCID: PMCPMC6218410.

109. Said S, Pazoki R, Karhunen V, Võsa U, Ligthart S, Bodinier B, et al. Genetic analysis of over half a million people characterises C-reactive protein loci. Nat Commun. 2022;13(1):2198. Epub 20220422. doi: 10.1038/s41467-022-29650-5. PubMed PMID: 35459240; PubMed Central PMCID: PMCPMC9033829.

110. Pazoki R, Vujkovic M, Elliott J, Evangelou E, Gill D, Ghanbari M, et al. Genetic analysis in European ancestry individuals identifies 517 loci associated with liver enzymes. Nat Commun. 2021;12(1):2579. Epub 20210510. doi: 10.1038/s41467-021-22338-2. PubMed PMID: 33972514; PubMed Central PMCID: PMCPMC8110798.

111. Raghavan S, Huang J, Tcheandjieu C, Huffman JE, Litkowski E, Liu C, et al. A multi-population phenome-wide association study of genetically-predicted height in the Million Veteran Program. PLoS Genet. 2022;18(6):e1010193. Epub 20220602. doi: 10.1371/journal.pgen.1010193. PubMed PMID: 35653334; PubMed Central PMCID: PMCPMC9162317.

112. Mahajan A, Taliun D, Thurner M, Robertson NR, Torres JM, Rayner NW, et al. Fine-mapping type 2 diabetes loci to single-variant resolution using high-density imputation and islet-specific epigenome maps. Nat Genet. 2018;50(11):1505-13. Epub 20181008. doi: 10.1038/s41588-018-0241-6. PubMed PMID: 30297969; PubMed Central PMCID: PMCPMC6287706.

113. Brandes N, Linial N, Linial M. Genetic association studies of alterations in protein function expose recessive effects on cancer predisposition. Sci Rep. 2021;11(1):14901. Epub 20210721. doi: 10.1038/s41598-021-94252-y. PubMed PMID: 34290314; PubMed Central PMCID: PMCPMC8295298.

114. Michailidou K, Hall P, Gonzalez-Neira A, Ghoussaini M, Dennis J, Milne RL, et al. Large-scale genotyping identifies 41 new loci associated with breast cancer risk. Nat Genet. 2013;45(4):353-61, 61e1-2. doi: 10.1038/ng.2563. PubMed PMID: 23535729; PubMed Central PMCID: PMCPMC3771688.

115. Rashkin SR, Graff RE, Kachuri L, Thai KK, Alexeeff SE, Blatchins MA, et al. Pan-cancer study detects genetic risk variants and shared genetic basis in two large cohorts. Nat Commun. 2020;11(1):4423. Epub 20200904. doi: 10.1038/s41467-020-18246-6. PubMed PMID: 32887889; PubMed Central PMCID: PMCPMC7473862.

116. Shu X, Long J, Cai Q, Kweon SS, Choi JY, Kubo M, et al. Identification of novel breast cancer susceptibility loci in meta-analyses conducted among Asian and European descendants. Nat Commun. 2020;11(1):1217. Epub 20200305. doi: 10.1038/s41467-020-15046-w. PubMed PMID: 32139696; PubMed Central PMCID: PMCPMC7057957.

117. Michailidou K, Beesley J, Lindstrom S, Canisius S, Dennis J, Lush MJ, et al. Genome-wide association analysis of more than 120,000 individuals identifies 15 new susceptibility loci for breast cancer. Nat Genet. 2015;47(4):373-80. Epub 20150309. doi: 10.1038/ng.3242. PubMed PMID: 25751625; PubMed Central PMCID: PMCPMC4549775.

118. Michailidou K, Lindström S, Dennis J, Beesley J, Hui S, Kar S, et al. Association analysis identifies 65 new breast cancer risk loci. Nature. 2017;551(7678):92-4. Epub 20171023. doi: 10.1038/nature24284. PubMed PMID: 29059683; PubMed Central PMCID: PMCPMC5798588.

119. Chen VL, Du X, Chen Y, Kuppa A, Handelman SK, Vohnoutka RB, et al. Genome-wide association study of serum liver enzymes implicates diverse metabolic and liver pathology. Nat Commun. 2021;12(1):816. Epub 20210205. doi: 10.1038/s41467-020-20870-1. PubMed PMID: 33547301; PubMed Central PMCID: PMCPMC7865025.

120. Yap CX, Sidorenko J, Wu Y, Kemper KE, Yang J, Wray NR, et al. Dissection of genetic variation and evidence for pleiotropy in male pattern baldness. Nat Commun. 2018;9(1):5407. Epub 20181220. doi: 10.1038/s41467-018-07862-y. PubMed PMID: 30573740; PubMed Central PMCID: PMCPMC6302097.

121. Kim SK. Identification of 613 new loci associated with heel bone mineral density and a polygenic risk score for bone mineral density, osteoporosis and fracture. PLoS One. 2018;13(7):e0200785. Epub 20180726. doi: 10.1371/journal.pone.0200785. PubMed PMID: 30048462; PubMed Central PMCID: PMCPMC6062019.

122. Tekola Ayele F, Doumatey A, Huang H, Zhou J, Charles B, Erdos M, et al. Genome-wide associated loci influencing interleukin (IL)-10, IL-1Ra, and IL-6 levels in African Americans. Immunogenetics. 2012;64(5):351-9. Epub 20111229. doi: 10.1007/s00251-011-0596-7. PubMed PMID: 22205395; PubMed Central PMCID: PMCPMC3418332.

123. Yang SK, Hong M, Oh H, Low HQ, Jung S, Ahn S, et al. Identification of Loci at 1q21 and 16q23 That Affect Susceptibility to Inflammatory Bowel Disease in Koreans. Gastroenterology. 2016;151(6):1096-9.e4. Epub 20160826. doi: 10.1053/j.gastro.2016.08.025. PubMed PMID: 27569725.

124. Hanscombe KB, Persyn E, Traylor M, Glanville KP, Hamer M, Coleman JRI, et al. The genetic case for cardiorespiratory fitness as a clinical vital sign and the routine prescription of physical activity in healthcare. Genome Med. 2021;13(1):180. Epub 20211109. doi: 10.1186/s13073-021-00994-9. PubMed PMID: 34753499; PubMed Central PMCID: PMCPMC8579601.

125. He W, Han X, Ong JS, Hewitt AW, Mackey DA, Gharahkhani P, et al. Association of Novel Loci With Keratoconus Susceptibility in a Multitrait Genome-Wide Association Study of the UK Biobank Database and Canadian Longitudinal Study on Aging. JAMA Ophthalmol. 2022;140(6):568-76. doi: 10.1001/jamaophthalmol.2022.0891. PubMed PMID: 35446358; PubMed Central PMCID: PMCPMC9026225.

126. White JD, Indencleef K, Naqvi S, Eller RJ, Hoskens H, Roosenboom J, et al. Insights into the genetic architecture of the human face. Nat Genet. 2021;53(1):45-53. Epub 20201207. doi: 10.1038/s41588-020-00741-7. PubMed PMID: 33288918; PubMed Central PMCID: PMCPMC7796995.

127. Chu X, Liu L, Ye J, Wen Y, Li P, Cheng B, et al. Insomnia affects the levels of plasma bilirubin and protein metabolism: an observational study and GWGEIS in UK Biobank cohort. Sleep Med. 2021;85:184-90. Epub 20210701. doi: 10.1016/j.sleep.2021.05.040. PubMed PMID: 34343768.

128. Yoshida T, Kato K, Yokoi K, Oguri M, Watanabe S, Metoki N, et al. Association of genetic variants with chronic kidney disease in Japanese individuals with or without hypertension or diabetes mellitus. Exp Ther Med. 2010;1(1):137-45. Epub 20100101. doi: 10.3892/etm_00000023. PubMed PMID: 23136606; PubMed Central PMCID: PMCPMC3490372.

129. Donati G, Dumontheil I, Pain O, Asbury K, Meaburn EL. Evidence for specificity of polygenic contributions to attainment in English, maths and science during adolescence. Sci Rep. 2021;11(1):3851. Epub 20210216. doi: 10.1038/s41598-021-82877-y. PubMed PMID: 33594131; PubMed Central PMCID: PMCPMC7887196.

130. Okbay A, Beauchamp JP, Fontana MA, Lee JJ, Pers TH, Rietveld CA, et al. Genome-wide association study identifies 74 loci associated with educational attainment. Nature. 2016;533(7604):539-42. Epub 20160511. doi: 10.1038/nature17671. PubMed PMID: 27225129; PubMed Central PMCID: PMCPMC4883595.

131. Wang H, Yang J, Schneider JA, De Jager PL, Bennett DA, Zhang HY. Genome-wide interaction analysis of pathological hallmarks in Alzheimer's disease. Neurobiol Aging. 2020;93:61-8. Epub 20200429. doi: 10.1016/j.neurobiolaging.2020.04.025. PubMed PMID: 32450446.

132. Homann J, Osburg T, Ohlei O, Dobricic V, Deecke L, Bos I, et al. Genome-Wide Association Study of Alzheimer's Disease Brain Imaging Biomarkers and Neuropsychological Phenotypes in the European Medical Information Framework for Alzheimer's Disease Multimodal Biomarker Discovery Dataset. Front Aging Neurosci. 2022;14:840651. Epub 20220321. doi: 10.3389/fnagi.2022.840651. PubMed PMID: 35386118; PubMed Central PMCID: PMCPMC8979334.

133. Alaraudanjoki VK, Koivisto S, Pesonen P, Männikkö M, Leinonen J, Tjäderhane L, et al. Genome-Wide Association Study of Erosive Tooth Wear in a Finnish Cohort. Caries Res. 2019;53(1):49-59. Epub 20180613. doi: 10.1159/000488208. PubMed PMID: 29898447.

134. Seshadri S, DeStefano AL, Au R, Massaro JM, Beiser AS, Kelly-Hayes M, et al. Genetic correlates of brain aging on MRI and cognitive test measures: a genome-wide association and linkage analysis in the Framingham Study. BMC Med Genet. 2007;8 Suppl 1(Suppl 1):S15. Epub 20070919. doi: 10.1186/1471-2350-8-s1-s15. PubMed PMID: 17903297; PubMed Central PMCID: PMCPMC1995608.

135. Davies G, Lam M, Harris SE, Trampush JW, Luciano M, Hill WD, et al. Study of 300,486 individuals identifies 148 independent genetic loci influencing general cognitive function. Nat Commun. 2018;9(1):2098. Epub 20180529. doi: 10.1038/s41467-018-04362-x. PubMed PMID: 29844566; PubMed Central PMCID: PMCPMC5974083.

136. Buniello A, MacArthur JAL, Cerezo M, Harris LW, Hayhurst J, Malangone C, et al. The NHGRI-EBI GWAS Catalog of published genome-wide association studies, targeted arrays and summary statistics 2019. Nucleic Acids Res. 2019;47(D1):D1005-d12. doi: 10.1093/nar/gky1120. PubMed PMID: 30445434; PubMed Central PMCID: PMCPMC6323933.
